# Hydrogels and Microgels: Driving Revolutionary Innovations in Targeted Drug Delivery, Strengthening Infection Management, and Advancing Tissue Repair and Regeneration

**DOI:** 10.3390/gels11030179

**Published:** 2025-03-03

**Authors:** Md. Shahriar Ahmed, Sua Yun, Hae-Yong Kim, Sunho Ko, Mobinul Islam, Kyung-Wan Nam

**Affiliations:** 1 Department of Energy & Materials Engineering, Dongguk University, Seoul 04620, Republic of Korea; shahriar.emcl@dgu.ac.kr (M.S.A.);; 2 Department of Advanced Battery Convergence Engineering, Dongguk University, Seoul 04620, Republic of Korea

**Keywords:** hydrogels, microgels, drug delivery, targeted delivery, infection management, antibacterial strategies, tissue repair

## Abstract

Hydrogels and microgels are emerging as pivotal platforms in biomedicine, with significant potential in targeted drug delivery, enhanced infection management, and tissue repair and regeneration. These gels, characterized by their high water content, unique structures, and adaptable mechanical properties, interact seamlessly with biological systems, making them invaluable for controlled and targeted drug release. In the realm of infection management, hydrogels and microgels can incorporate antimicrobial agents, offering robust defenses against bacterial infections. This capability is increasingly important in the fight against antibiotic resistance, providing innovative solutions for infection prevention in wound dressings, surgical implants, and medical devices. Additionally, the biocompatibility and customizable mechanical properties of these gels make them ideal scaffolds for tissue engineering, supporting the growth and repair of damaged tissues. Despite their promising applications, challenges such as ensuring long-term stability, enhancing therapeutic agent loading capacities, and scaling production must be addressed for widespread adoption. This review explores the current advancements, opportunities, and limitations of hydrogels and microgels, highlighting research and technological directions poised to revolutionize treatment strategies through personalized and regenerative approaches.

## 1. Introduction

Hydrogels are three-dimensional networks formed by cross-linked polymer chains with an impressive ability to absorb water, making them particularly valuable in the biomedical field. Fmoc amino acids are valuable in biomedical applications due to their ability to self-assemble into various structures, like hydrogels, through the inherent hydrophobicity and aromaticity of the Fmoc group, which allows for the design of functional biomaterials with potential uses in drug delivery, cell culture scaffolds, antibacterial applications, and more, all while maintaining good biocompatibility when carefully chosen and modified [1].

**Scheme 1:**
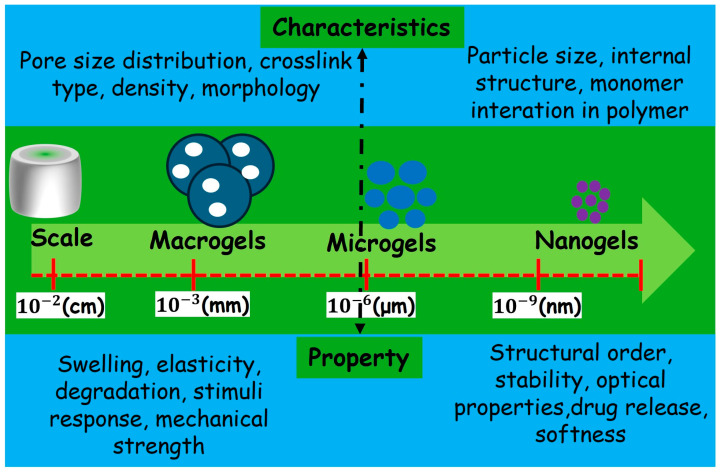
Structure–property relationship of macrogels, microgels, and nanogels formed at varying scales.

Their adaptable properties and unique behavior make them ideal for creating a wide array of materials, including contact lenses, blood-compatible hydrogels, scaffolds, wound-healing bioadhesives, artificial kidney membranes, artificial skin, vocal cord replacements, and artificial tendons. This versatility and significance make hydrogels a focal point of research [2]. In pharmaceutical applications, the porous structure of hydrogels is advantageous for drug delivery systems, as they can encapsulate active agents and release them at a controlled rate through swelling or disintegration, among other methods. Designing hydrogels to meet specific application requirements is facilitated by the wide range of polymers. This allows for control over properties like biodegradation rate, mechanical strength, swelling capacity, and responsiveness to external stimuli by combining natural and synthetic polymers [3]. The cross-linking process, crucial for creating these systems, can be accomplished through various methods, including radical reactions involving monomers and oligomers, as well as initiating reactions using ionizing radiation or UV light to excite functional groups. Additionally, chemical cross-linkers, which contain multiple functional groups for polymerization, offer another method to establish the network structure. While these techniques produce chemically cross-linked hydrogels, physical hydrogels can be formed through intermolecular interactions such as ionic, hydrophobic, and hydrogen bonding [4]. As the healthcare industry increasingly demands more advanced biomaterials, researchers are focusing on developing hydrogels derived from natural biopolymers [5,6]. These biomaterials can actively participate in therapeutic interventions, facilitating desired healing effects. Potential biopolymer sources include bacteria, animals, plants, and algae, each offering unique polysaccharides, polypeptides, or polynucleotides [7,8]. Depending on their origin and intended use, these biopolymers are extracted through specific chemical methods that substantially influence their chemical and physical properties [9,10]. Hydrogels crafted from these biopolymers aim to replicate the original characteristics of the body’s tissues, making them suitable for applications like soft tissue repair, tissue engineering, and drug delivery [11] Despite their promise, integrating these hydrogel systems to consistently respond to the body’s physiological and pathological variations remains a challenge. Therefore, advancing hydrogel scaffold technologies is crucial to facilitating effective tissue regeneration and expanding their use in biomedical applications. Hydrogels can be categorized based on particle size into macrogels, microgels, or nanogels. Particles smaller than 100 nm are typically classified as nanogels, while those that are larger, up to the micrometer range, are referred to as microgels (shown in Scheme 1). Micro- and nanostructures differ from bulk gels in that they operate like macromolecules with the ability to rapidly respond to external stimuli, thus earning them the classification of functional or smart materials. Furthermore, hydrogel substrates can be grafted onto various surfaces to improve their functionality and versatility [12]. The outstanding performance of such systems stems from their distinctive architecture, noted for being dynamic, permeable, and deformable [13]. These features are particularly useful for increasing stability in the transportation of biopharmaceuticals, such as proteins [14].

Hydrogels, both natural and synthetic, play a pivotal role in the advancement of regenerative medicine by influencing human stem cell differentiation. These hydrophilic polymer networks closely mimic the extracellular matrix, offering a conducive environment for stem cell growth and maturation. Natural hydrogels, derived from biological sources, possess inherent biocompatibility and bioactivity, providing biochemical cues that are essential for guiding stem cell fate. They can facilitate interactions that are vital for promoting specific lineage differentiation due to their similarity to native tissues. Conversely, synthetic hydrogels offer unparalleled control over physical and chemical properties, allowing precise manipulation of mechanical strength, degradation rates, and functionalization with bioactive signals. This versatility enables researchers to create customized microenvironments tailored to direct stem cell differentiation pathways. The integration of both hydrogel types can result in composite systems that leverage the strengths of each—natural hydrogels for their biological relevance and synthetic ones for their tunability and structural integrity In recent years, advances in hydrogel technology have enabled precise control over the microenvironmental cues that influence stem cell behavior, such as stiffness, porosity, and the presence of biochemical signals. This has opened up new possibilities for creating more accurate models of tissue development and regeneration, as well as for designing innovative therapeutic strategies for tissue engineering and repair. The versatility of hydrogels allows them to serve as delivery vehicles for bioactive molecules that promote specific lineage commitments, thus enhancing the efficiency of stem cell differentiation into desired cell types. Furthermore, their biocompatibility and tunability have made hydrogels a platform for integrating various elements of the cellular microenvironment, which is crucial for achieving functional tissue architecture in vitro and in vivo.

### 1.1. Natural and Synthetic Hydrogels

Chitosan-based hydrogels offer promising avenues in biological applications due to their biocompatibility and bioactivity. These hydrogels find utility in tissue engineering, wound healing, and drug delivery systems, providing a versatile platform for controlled release and cell interaction, thus advancing therapeutic interventions in regenerative medicine. Chitosan-based hydrogels have garnered significant attention for their inherent antibacterial properties, primarily due to their positively charged characteristics. Recent studies have highlighted the development of advanced chitosan (CS)-based composite hydrogels that exhibit both enhanced mechanical performance and potent antibacterial efficacy. For instance, Zhu Honglin et al. [15] created a series of double-network hydrogels that demonstrated impressive antibacterial inhibition rates of approximately 72.85% against *Listeria monocytogenes* and 76.60% against *E. coli*. Additionally, an injectable chitosan-based hydrogel with self-healing and wound-healing capabilities was formulated by Deng Pengpeng et al. [16]. This hydrogel was developed through an amidation reaction, where the amine groups of chitosan reacted with carboxyl adenine groups, followed by a controlled heating and cooling process to form the hydrogel. The resulting material showcased remarkable antibacterial properties, achieving effectiveness rates of about 95.3% against *E. coli*, 97.4% against *Staphylococcus aureus*, and complete inhibition (100%) of *Candida albicans*. The continued exploration of natural polymeric hydrogels, such as chitosan, underscores their potential for diverse antibacterial applications in biomedical fields. Tannic acid (TA) is an amphiphilic tannin present in various natural sources, characterized by its otriphenol and catechol moieties, which provide noteworthy antioxidant and antibacterial properties against Gram-positive and Gram-negative bacteria. The abundant hydroxyl and carboxyl groups in TA enable versatile interactions with synthetic compounds, facilitating the development of hydrogels via multi-cross-linking techniques. Sahiner et al. [17] demonstrated the synthesis of a linear polyethyleneimine (PEI)-based hydrogel utilizing TA as an active antibacterial agent. Their research identified a minimum bactericidal concentration (MBC) of TA against *E. coli* and *Bacillus subtilis* as low as 5 mg/mL, highlighting its promising potential for incorporation into synthetic hydrogels aimed at antibacterial applications in biomedical fields. Natural polymeric antibacterial hydrogels are well-suited for wound healing; however, their commonly weak mechanical properties in aqueous solutions limit their practical applications. While they can disrupt bacterial structures through electrostatic interactions with membrane proteins and phospholipids, these hydrogels often lack selectivity and long-term efficacy. Therefore, further research is required to enhance their specificity and stability against bacteria, which is essential for improving their clinical effectiveness in wound management. Synthetic hydrogels are polymeric materials designed through chemical processes to possess specific properties tailored for diverse biomedical applications. Their tunability enables precise control over aspects like swelling behavior, mechanical strength, and degradation rates, making them highly versatile. In drug delivery systems, synthetic hydrogels can encapsulate therapeutic agents and provide controlled release, enhancing treatment efficacy while minimizing side effects. They are also utilized as scaffolds in tissue engineering, supporting cell growth and the regeneration of various tissues, such as cartilage and skin. Additionally, these hydrogels serve as effective wound dressings, offering moisture retention and protection against infections. Overall, the adaptability and functional capabilities of synthetic hydrogels position them as vital components in advancing modern biomedical technologies. Among the various synthetic polymer-linked hydrogels, poly (ethylene glycol) (PEG) stands out as a significant candidate due to its exceptional properties. Recent explorations have illuminated the increasing interest in PEG-based hydrogels and their derivatives, especially regarding their favorable biocompatibility in drug delivery and tissue engineering applications. A notable advantage of these materials is the emergence of injectable, in situ-gelling formulations, which allow for non-invasive administration via low-viscosity precursor polymer solutions, thereby broadening their potential applications [18].

### 1.2. Mechanical Characterization of the Hydrogels

Hydrogel biomaterials are fundamental components in the construction of engineered tissues, with significant applications in regenerative medicine and drug delivery systems. Their mechanical properties are crucial in mediating cell–extracellular matrix interactions and guiding cellular phenotype and genotype outcomes. Although there have been substantial advancements in methods and techniques to fine-tune the biomechanical properties of hydrogels, challenges remain. These include difficulties in synthesizing hydrogels with intricate mechanical characteristics and limitations in achieving effective vascularization and patterning of the complex structures reminiscent of natural tissues. These barriers hinder the development of sophisticated, fully functional organs. Mechanical characterization of hydrogels is essential to assess their applicability in biomedical fields such as tissue engineering and drug delivery, where understanding parameters like stiffness, porosity, and degradation rate is critical. Stiffness, often described by the elastic modulus, is crucial for influencing cellular responses; it can be adjusted through the composition and crosslinking density of the hydrogel, allowing for the mimicry of the mechanical properties of various tissues, thereby providing a conducive environment for specific cellular functions like osteogenic differentiation in bone tissue engineering.

Shih Jye Tan et al. [19] explored the role of mechanical stimuli in the matrix to create materials that mimic physiological environments, thus supporting cell growth and differentiation. They investigated transglutaminase cross-linked gelatin (TG-Gel), showing that its mechanical properties can be tailored by adjusting gelatin concentration, affecting cellular responses like gene expression and differentiation. Stiffer TG-Gels enhanced focal contact formation and osteogenic differentiation, while softer gels promoted proliferation. The study also examined how matrix rigidity and BMP-2 interact synergistically to enhance osteogenesis, suggesting optimized scaffold designs for bone regeneration.

Porosity plays a fundamental role in nutrient exchange, cell infiltration, and vascularization, with its optimization critical for successful tissue integration and regeneration. Similarly, the degradation rate of hydrogels must be finely tuned to ensure they degrade commensurately with new tissue growth, thus providing structural support while preventing negative inflammatory responses. This can be managed through hydrolytic or enzymatic pathways tailored to specific applications, such as controlled drug release systems that respond to physiological stimuli. Injectable and porous biomaterials present promising opportunities for bioactive delivery and regenerative medicine. Porosity is crucial for applications that require cell infiltration or nutrient distribution throughout the scaffold. However, many conventional methods to create porosity in hydrogels involve cytotoxic substance extraction techniques, making them unsuitable for injectable systems. Numerous studies have been conducted to develop methods for inducing porous structures specifically in injectable hydrogels [20].

### 1.3. Effect of Hydrogel Stiffness on Stem Cell Behavior

In recent years, the exploration of hydrogel stiffness has become a focal point in tissue engineering due to its significant impact on cell behavior. Hydrogels, as bio-scaffold materials with excellent biocompatibility and biodegradability, offer a versatile platform where stiffness can be modulated by altering crosslinking methods, applying external stimuli, or adjusting material composition, including the addition of nanoparticles [21]. The effect of different stiffness levels on stem cells, such as mesenchymal stem cells (MSCs), fibroblasts, and macrophages, underscores the potential of hydrogels to influence tissue repair and regeneration. Stiffer hydrogels have been shown to promote osteogenic differentiation in Mesenchymal Stem Cells (MSCs), making them ideal for applications in bone regeneration, whereas softer matrices encourage differentiation into chondrocytes or adipocytes, highlighting the need to match gel stiffness with target tissue requirements [22]. Furthermore, hydrogel stiffness also affects macrophage phenotype, subsequently altering the tissue’s inflammatory environment and promoting angiogenesis, which are critical factors in effective wound healing. The cellular response to hydrogel stiffness involves changes in the expression of proteins and oxidative stress levels, influencing cell activities such as differentiation, migration, and adhesion. Recent advances have even introduced hydrogels with gradient stiffness, which significantly impact tissue repair at cartilage–bone interfaces [23]. Importantly, the dimensionality of hydrogel systems (2D vs. 3D) plays a crucial role in replicating tissue environments. Most studies have traditionally used 2D hydrogels, but the development of 3D hydrogels with adjustable stiffness has provided insights that more accurately reflect vivo conditions [24]. These 3D systems maintain the relevance of physical cues, driving cell proliferation and differentiation as observed models mimicking skin and muscle tissues. The ability to fine-tune hydrogel stiffness offers tremendous potential in tissue engineering and regenerative medicine by shaping cell behaviors to promote desired differentiation pathways and enhance tissue repair.

Gels are categorized by their origin, preparation methods, crosslinking types, swelling characteristics, ionic charges, stimuli responsiveness, and biodegradability [25,26]. Hydrogels display diversity in size, structure, architecture, and properties, all of which influence their functionality and potential applications [27,28]. The viability of hydrogel applications hinges on certain properties such as size, swelling capacity, viscosity, elasticity, rigidity, and mechanical strength, alongside their chemical composition and structure. These properties are determined by the hydrogel’s structural features, including polymer dimensions, orientation and composition, crosslink density (mesh size), network architecture, water content (either bound or free), and the strength of the chemical bonds involved [29].

Shape-memory polymer-based hydrogels are advanced materials that combine the water-absorbing properties of hydrogels with shape memory functionality. These materials can transition between temporary and original shapes when exposed to stimuli like temperature or pH changes. Made from stimuli-responsive polymers, these hydrogels have applications in biomedical devices, drug delivery, and tissue engineering. They offer flexibility and adaptability, making them ideal for minimally invasive surgical tools, responsive drug release, and dynamic scaffolds, thus expanding the potential of traditional hydrogel systems. For example, composite biomaterials made by integrating microgels into collagen-1 networks exhibit reversible stiffening and shaping capabilities suited for biomedical scaffolds. The materials show strain stiffening and can hold deformed shapes without external stress. Our analysis aligns their rheological behavior with models of cellular mechanics, demonstrating the potential for adaptable and form-fitting biomedical applications [30].

Microgels, which range in size from centimeters to millimeters, feature a macroscopic network structure. They often reach a gel point beyond which they can divide into gel and sol components [31,32]. Despite having similar polymer chemistry and thermodynamic characteristics, such as crosslink density distribution and phase transition behavior, macrogels and microgels differ in their physical molecular arrangements. Additionally, microgels, or hydrogel beads, microspheres, biopolymer particles, with nanogels referring to sizes under 100 nm [33,34], are smaller discrete particles ranging from approximately 100 nm to 100 μm in size. These microgels consist of a three-dimensional network of crosslinked polymer chains capable of hosting a large volume of solvent [35]. This classification represents systems where gelation occurs in constrained dimensions, with the confines lifted after crosslinking where microgels possess precisely controlled mechanical and chemical properties, achievable through both top-down or bottom-up preparation methods [36]. They exhibit distinctive functional and dynamic characteristics, such as differential swelling, a large surface area, and a permeable and deformable structure. Additionally, their morphology and architecture can be tailored as needed. These properties make microgels suitable for various applications, including biocatalysis, biosensors, biological scavenging, drug delivery with precision, delivery of macromolecular therapeutics, multidrug release, tissue engineering, and the development of bio-microdevices [37].

In this review, we are examining the fundamental differences, synthesis properties, and diverse applications of hydrogels and microgels in biomedical engineering and pharmaceuticals. We will also consider future directions in the field and emerging research is likely to focus on enhancing the specificity and efficiency of these materials for targeted drug delivery, developing smart materials with improved responsiveness to external stimuli, and expanding their use in tissue engineering. We also represent the advances in fabrication techniques and the integration of bioactive components, which are expected to further broaden the scope and functionality of these gels, paving the way for innovative applications in personalized medicine and regenerative therapies.

## 2. Synthesis of Hydrogel

Hydrogels, known for their vast water absorption capacity and biocompatibility, are a fascinating class of materials with diverse applications ranging from biomedical engineering to environmental remediation. These three-dimensional, hydrophilic polymer networks can be prepared through a variety of methods that influence their physical and chemical properties, thus tailoring them for specific uses. Hydrogels are structured from polymer chains, and their overall characteristics largely depend on the specific properties of these polymers. One of the hallmark features of hydrogels is their capacity to absorb water, but not all polymers are conducive to creating effective hydrogels. In their structure, polymer chains are interconnected through cross-linking, forming a three-dimensional network that plays a crucial role in determining the physical attributes of the hydrogel [38]. Cross-linking contributes to an increase in the effective molecular weight of the polymer chains while simultaneously restricting their movement, which leads to a reduction in solubility. Despite their insolubility, these cross-linked polymers can take up significant amounts of solvent, resulting in a gel-like appearance. The extent to which a polymer network can absorb liquid is influenced by the cross-linking density, which refers to the number of cross-links within a given volume. As cross-linking density rises, the flexibility of the polymer chains to interact with solvent molecules decreases. Within hydrogels, cross-links can manifest as either physical or chemical bonds. The process of cross-linking can take place during the concurrent growth of polymer chains or after the chains have fully formed. This flexibility in the preparation process means hydrogels can be synthesized starting from monomers, prepolymers, or existing polymers [39]. Understanding these fundamental aspects of hydrogel chemistry is essential for customizing their properties to suit a wide array of applications, including biomedical, environmental, and industrial uses. The preparation of hydrogels can be broadly categorized into physical and chemical methods. Chemical cross-linking involves covalent bond formation between polymer chains, often initiated through reactions with cross-linking agents or by employing radiation techniques like gamma rays or UV light [40]. This method is renowned for producing stable hydrogels with uniform properties.

### 2.1. Physically Cross-Linked Hydrogels

Furthermore, physical cross-linking relies on non-covalent interactions, such as ionic bonding, hydrogen bonding, or hydrophobic interactions. Methods such as freeze–thaw cycles or ionic cross-linking using multivalent ions are common, offering the advantage of avoiding potentially harmful cross-linking agents, making them ideal for certain biomedical applications. Physically cross-linked hydrogels are a fascinating class of materials characterized by their construction through non-covalent interactions, such as hydrogen bonding, self-assembly, and crystallization. Unlike chemically cross-linked hydrogels, these physical interactions provide reversible and tunable bonding, allowing hydrogels to respond to environmental changes, which is particularly advantageous in applications where biocompatibility and stimuli-responsiveness are desired. Hydrogen bonding within these hydrogels typically involves interactions between molecules that possess nitrogen–hydrogen (N-H), oxygen–hydrogen (O-H), and fluorine–hydrogen (F-H) functionalities. An illustrative example is observed in polyethylene glycol (PEG), where the oxygen atoms form hydrogen bonds with carboxylic acid groups found in poly (acrylic acid) (PAA), enhancing its network structure [41]. These interactions create a dynamic and flexible hydrogel network that can be finely tuned based on the specific functional groups involved. Crystallization, another key mechanism for physical cross-linking, involves the organization of polymer chains into crystalline regions, increasing the material’s structural integrity. This is exemplified in the synthesis of poly (vinyl alcohol) (PVA) hydrogels, where freeze–thaw cycles induce crystallization, resulting in strong and resilient hydrogels [42]. This method enhances the mechanical properties of hydrogel without the need for chemical cross-linkers, making it a safer alternative for various biomedical applications. To fabricate physically cross-linked hydrogels, a range of methods can be employed. These include the careful heating or cooling of polymer solutions to promote or inhibit crystallization, the strategic use of freeze–thaw cycles to control crystallization and network formation, adjusting the pH to modulate ionic interactions, and mixing polymers with complementary ionic charges. The latter involves combining polyanions and polycations or introducing a polyelectrolyte solution to a multivalent ion of opposite charge, which can significantly enhance the hydrogel’s network stability and responsiveness [43]. The versatility and adaptability of physically cross-linked hydrogels make them an attractive choice for a broad spectrum of applications, from drug delivery and tissue engineering to environmental management and industrial uses, where their ability to respond to stimuli and self-heal can be particularly beneficial.

### 2.2. Chemically Cross-Linked Hydrogels

**Scheme 2:**
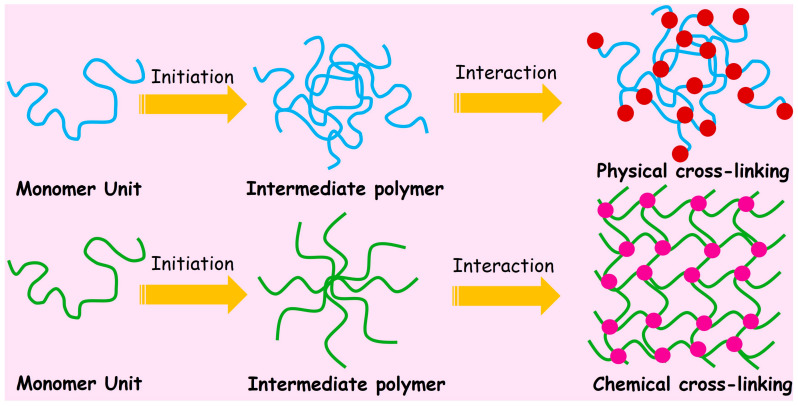
Schematic representation of physical and chemical cross-linking between polymer chains in a hydrogel.

Chemically cross-linked hydrogels are structured through the creation of covalent bonds, resulting in a stable and often permanent network. Several techniques can be employed to achieve such networks, including copolymerization with multifunctional monomers, exposure to high-energy radiation, and reactions involving complementary or pendant groups, as well as the use of specialized cross-linking agents [44,45]. In bulk polymerization, radical initiators are dissolved directly in the liquid monomer, whereas solution polymerization involves a solution containing monomers, a cross-linking agent, and an initiator. Initiation is typically activated by UV radiation, temperature increases, or a redox process. High-energy radiation is particularly effective in inducing radical formation along polymer chains, which then link together to create a cohesive network. This method is commonly used for producing hydrogels such as poly (vinyl alcohol) (PVA), polyethylene glycol (PEG), and poly (acrylic acid) (PAA) through irradiation [40,46]. In addition to traditional radical polymerization, innovative chemical strategies, such as click chemistry and Schiff base reactions, are employed to craft chemically cross-linked hydrogels. These methods are valued for their speed, versatility, and precision; click chemistry provides high efficiency and specificity while avoiding the generation of byproducts [47]. These chemical cross-linking approaches result in hydrogels with remarkable mechanical properties, stability, and potential for customization, making them exceptionally suitable for applications ranging from drug delivery systems and tissue engineering scaffolds to various industrial uses. These features ensure that chemically cross-linked hydrogels remain a critical component in advancing material science and engineering. Another innovative approach is reverse thermal gelation, where polymers transition from a solution to a gel upon temperature change. This temperature-sensitive property is particularly useful for injectable hydrogels in drug delivery and tissue engineering. In addition to these methods, advanced techniques like electrospinning and microfluidics are being explored to create hydrogels with complex structures or micro-sized dimensions, expanding their applicability in diverse fields. Furthermore, the development of smart hydrogels that respond to environmental stimuli such as pH, temperature, or light has opened new horizons in targeted therapy and biosensing [48,49]. Both physical and chemical cross-links are represented in Scheme 2.

## 3. Biomedical Applications of Hydrogels

Hydrogels have become a cornerstone in the field of biomedical applications, notably in areas such as tissue engineering, drug delivery, and the development of medical devices, due to their unique ability to replicate the natural extracellular matrix (ECM) of human tissues. This replication provides a supportive environment for cell growth and differentiation, making hydrogels an ideal scaffold for tissue repair and regeneration. However, traditional static hydrogels are limited in their functionality because they do not possess the capability to dynamically adjust to environmental changes. This limitation restricts their effectiveness in withstanding and adapting to the complex and ever-changing biophysical microenvironments found in the human body, as well as in achieving on-demand tasks such as precise drug release and mechanical modifications. In contrast, the latest advancements have brought forth multifunctional dynamic hydrogels that can respond to various external stimuli, such as temperature, pH, light, and magnetic or electric fields. These dynamic hydrogels have captured significant attention in recent research due to their enhanced capability to perform specialized tasks and improve therapeutic outcomes. One of the most promising developments in this area is the incorporation of nanomaterials into these hydrogels. This integration not only enhances their mechanical strength and durability but also imbues hydrogels with new functionalities that are essential for a variety of advanced biomedical applications. For instance, in targeted drug delivery, nanomaterial-enhanced dynamic hydrogels can be engineered to release therapeutic agents in a controlled manner when triggered by specific physiological conditions, thereby optimizing treatment efficacy and minimizing side effects. Similarly, in wound healing, these hydrogels can respond to the wound environment, facilitating accelerated healing through the gradual release of growth factors or antimicrobial agents. Moreover, dynamic hydrogels also show great potential in the development of responsive medical implants that adjust to bodily changes, enhancing patient comfort and implant performance. The integration of nanotechnology with dynamic hydrogels has thus opened a wealth of new possibilities, enabling applications that were previously unattainable with conventional hydrogels, and paving the way for future innovations in personalized medicine and regenerative therapies.

### 3.1. Wound Dressings

**Figure 1:**
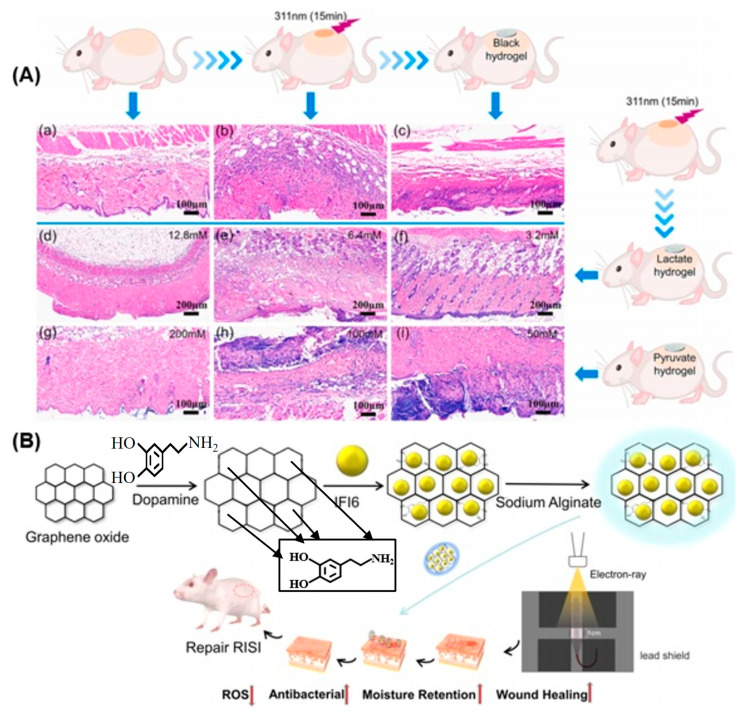
(**A**) Images of BALB/c mouse skin subjected to UV irradiation and treated with composite hydrogel films: (**a**) normal skin, (**b**) skin irradiated with 311 nm UV light, (**c**) hydrogels lacking pyruvate and lactic acid, (**d**–**f**) hydrogels containing varying concentrations of lactate (12.8, 6.4, and 3.2 mM), and (**g**–**i**), and hydrogels formulated with different levels of pyruvate (200, 100, and 50 mM); reproduced with permission [50], Elsevier. (**B**) Schematic diagram detailing the fabrication of IFI_6_-PDA@GO/SA and its role in healing radiation-induced skin injuries (RISIs) [51].

Hydrogels have become an integral component of skincare formulations due to their outstanding hydrating capabilities and ability to deliver active ingredients effectively. Their unique three-dimensional network structure allows them to retain a large volume of water, providing deep and sustained hydration to the skin, which is particularly beneficial for addressing dryness and irritation. In skincare products, hydrogels are utilized in a variety of applications, including moisturizing masks that adhere closely to the skin for enhanced ingredient penetration and to provide a calming effect. They are also employed in eye patches, which help reduce puffiness and dark circles by delivering revitalizing compounds such as peptides and botanical extracts around the eyes. Additionally, hydrogels are found in acne treatments, offering targeted delivery of anti-inflammatory and antibacterial agents to mitigate blemishes while maintaining skin moisture. Their cooling properties make hydrogels ideal for after-sun products, soothing sun-exposed skin and restoring lost moisture. By incorporating hydrogels, skincare products can achieve greater effectiveness in delivering moisture and beneficial compounds, catering to diverse skincare needs and enhancing overall skin health. In 2023, M. Liu and colleagues investigated hydrogel films composed of pyruvate and lactate for mitigating UV-induced skin inflammation and oxidative stress. This research aimed to utilize these compounds within hydrogels as a topical remedy for solar dermatitis, addressing both free radical scavenging and inflammation modulation. The study provided detailed schematics of the gel components and application process on UV-damaged skin, confirming the effectiveness of the lactate and pyruvate combination for treating UV-induced photodamage. Experimental results from a UV-irradiated BALB/c mouse skin injury model treated with the composite hydrogel film demonstrated reduced inflammation, as fewer inflammatory cells were present with varying concentrations of lactate (12.8, 6.4, and 3.2 mM) and pyruvate (200, 100, and 50 mM), highlighting their therapeutic potential through a self-tissue-repairing mechanism (as shown in Figure 1A) [50]. Additionally, another promising application involved IFI6 in promoting the healing of radiation-induced skin injuries (RISIs) through the modulation of HSF1 activity. A sprayable composite hydrogel containing IFI_6_-PDA@GO/SA was developed and applied to HaCaT skin cells, enhancing cell proliferation and migration and providing synergistic radio resistance both in vitro and in vivo. The study also evaluated the biological activity of IFI6 in wound healing through these hydrogels by assessing cell proliferation, migration, and angiogenesis. This research underscores the significant potential of IFI_6_-based treatments in managing RISI, advocating for further exploration of its broader therapeutic applications (as shown in Figure 1B) [51,52,53].

A gelatin-based antibacterial hydrogel is a versatile biomaterial designed for skin applications, particularly in wound healing. It uses gelatin, a biocompatible and biodegradable polymer, as a foundation to create a moist wound environment that promotes tissue regeneration. The hydrogel is enhanced with antibacterial agents, such as silver nanoparticles, chitosan, or essential oils, which provide broad-spectrum antimicrobial properties to prevent infections. This system allows for a controlled, sustained release of these agents, ensuring prolonged antibacterial action. Overall, these hydrogels are an effective solution for improving wound care by facilitating healing while protecting against microbial threats.

**Figure 2:**
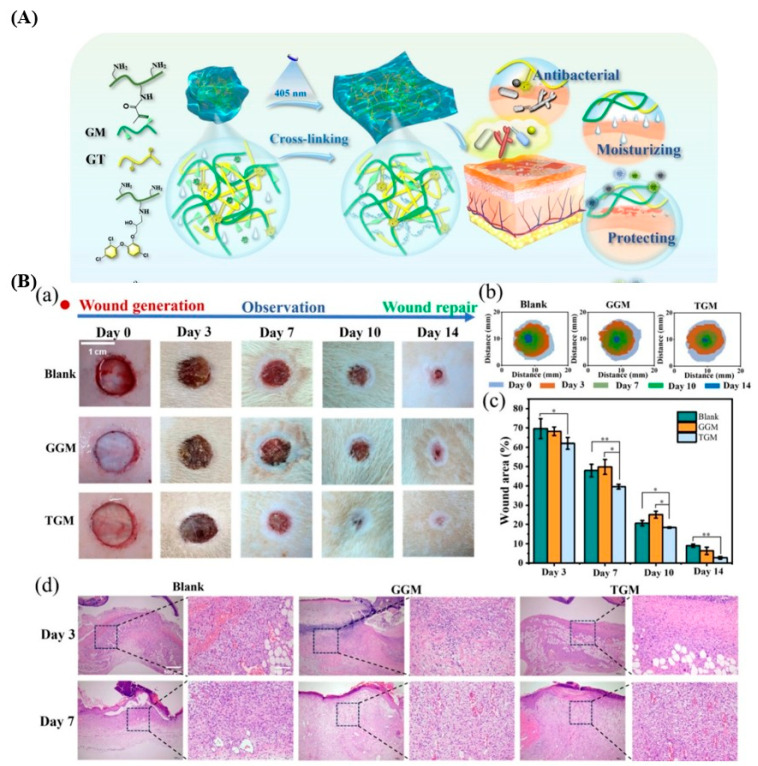
(**A**) Preparation and application of a gelatin-based antibacterial hydrogel (**B**). (**a**) Images showing wounds treated with various dressings over time; (**b**) diagram of wound sizes at days 0, 3, 7, 10, and 14; (**c**) wound area analysis at each time point for each group (* *p* < 0.05; ** *p* < 0.01); (**d**) H&E staining images of wounds on days 3 and 7; reprinted with permission from [54]. Copyright © 2023, American Chemical Society.

The synthesized antibacterial hydrogel, incorporating triclosan-grafted gelatin and photo-cross-linkable methacrylate gelatin, demonstrated exceptional antimicrobial efficacy when activated by visible blue light [54]. It achieved sterilization rates of 99.998% against both *E. coli* and *S. aureus*, 99.19% against *T. rubrum*, and 99.64% against *C. albicans*. Additionally, the hydrogel exhibited excellent biocompatibility, as evidenced by low cytotoxicity, minimal hemolysis, lack of skin irritation, and effective facilitation of wound healing in rats. These results indicate that this multifunctional hydrogel is a promising candidate for skincare applications, combining potent antimicrobial properties with safety for biological tissues, as shown in Figure 2A,B.

#### Dressing for Burn Wounds

Hydrogels are recognized as both safe and effective in the management of burn wounds, adaptable for use at every stage of treatment. As a rapidly advancing category of dressings, their unique structure makes them ideal carriers for active ingredients such as antimicrobials, agents that promote wound healing, biological agents, and growth factors. Although many hydrogel dressings have been evaluated using animal models, there is a pressing need for multicenter clinical studies to fully assess their true efficacy in clinical settings. The process of skin wound healing is complex, and using improper treatment methods can impede recovery. As such, there is a growing focus on methods for effective skin wound repair. Hydrogel dressings have emerged as one of the most promising materials for treating minor epidermal injuries due to their superior physical and chemical properties. Additionally, these hydrogels have applications in treating severe deep skin injuries, serving as a substitute for skin grafting by facilitating the regeneration and reconstruction of blood vessels and skin appendages. Despite ongoing research into hydrogels for skin wound repair, significant challenges remain in this field. Recently, advances in polymer chemistry have accelerated the development of skin tissue engineering. Based on their material sources, hydrogels are categorized into natural polymer-based, synthetic polymer-based, hybrid, and biomimetic polymer-based types.

**Figure 3:**
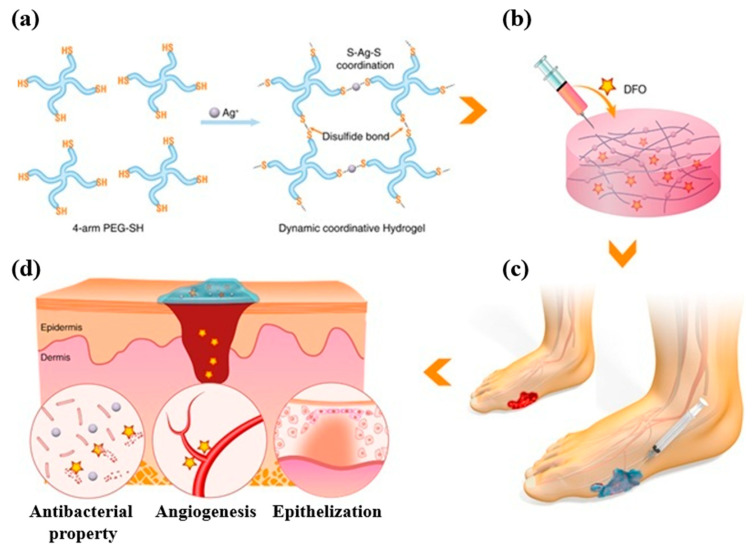
(**a**) Illustration of a self-healing hydrogel created by coordinating silver ions with thiol groups, using 4-arm-PEG-SH and AgNO_3_. (**b**) Multifunctional hydrogel encapsulates injectable drugs for repairing diabetic skin wounds. (**c**) Comparison of diabetic foot ulcers before and after hydrogel treatment. (**d**) Overview of how the injectable hydrogel aids skin repair [60].

Natural polymers, such as polysaccharides and proteins, are readily available and easy to source, and their excellent biocompatibility and biodegradability make them popular in skin tissue engineering applications. However, due to their inherently weak mechanical properties, these polymers often require enhancements through techniques like grafting, multiple crosslinking, or blending with other materials to improve their strength [55]. Hydrogel dressing can be achieved by functionalizing a double-network chitosan/alginate hydrogel with collagen peptides, thereby boosting both its mechanical properties and biological activity. Carboxymethyl chitosan, a water-soluble chitosan derivative known for its water retention capacity, was used by Cao et al. [56] alongside collagen to formulate a hydrogel that closely resembles the human extracellular matrix, offering essential nutrients for cell growth and proliferation. Gelatin, derived from the partial hydrolysis of collagen, is frequently used in wound dressings. However, its capacity for water absorption and mechanical strength can be inadequate. To overcome these limitations, Liu et al. [57] combined gelatin with poly (γ-glutamic acid) (γ-PGA) to produce hydrogels that not only absorb body fluids effectively but also expedite wound closure. Synthetic polymers, such as polyethylene glycol, polydopamine, polyacrylamide, and polyvinyl alcohol, present distinct benefits for skin tissue engineering due to their adjustable molecular weights and diverse properties [58]. These materials typically offer superior mechanical strength and more controlled biodegradation rates compared to natural polymers. Polyethylene glycol is particularly valued for its biocompatibility and water solubility, serving effectively in drug and nanoparticle delivery systems. For instance, a PEG-based hydrogel was created with thioketal bonds that respond to reactive oxygen species, making it suitable for delivering epidermal growth factors [59]. Vascular regeneration is essential for effective nutrient delivery and oxygen exchange during skin repair. Chen et al. [60] formulated an injectable hydrogel using four-arm thiolate polyethylene glycol (SH-PEG) and silver nitrate (AgNO_3_) through coordinated cross-linking, incorporating the angiogenic agent deferoxamine (DFO). This hydrogel effectively promoted angiogenesis, enhancing vascular network formation and extending blood vessel length, while the presence of DFO enhanced the antibacterial efficacy of silver ions (Ag^+^) (shown in Figure 3).

**Figure 4:**
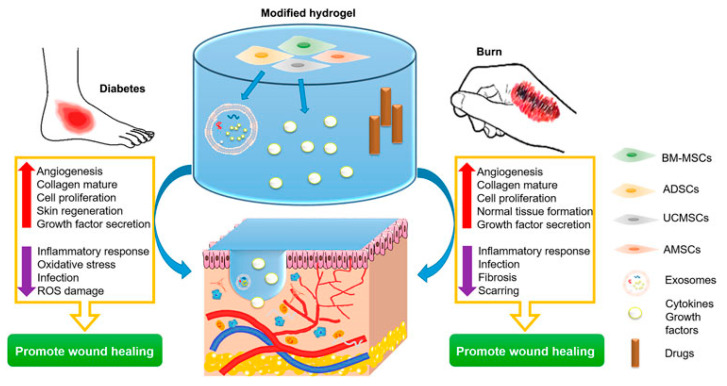
An approach using hydrogel stem cell technology for the treatment of diabetic wounds and burns [66].

In a recent study, researchers embarked on a journey to explore the potential benefits of using hydrogel dressing as an additional pain relief aid in first aid for children suffering from acute burn injuries. This investigation was structured as a prospective, randomized controlled trial, where young participants were divided into two distinct groups. One group received an inert hydrogel dressing, while the other was treated with a traditional polyvinyl chloride film as standard care. As the study progressed, the researchers meticulously assessed the pain scores of 17 pediatric burn patients. Surprisingly, the conclusion of this compassionate and thorough inquiry revealed no significant difference in pain relief between the children treated with hydrogel dressings and those who received conventional care, painting a nuanced picture of the effectiveness of hydrogels in managing acute pediatric burn pain [61]. In a series of studies examining the effectiveness of hydrogel dressings in burn wound care, several key findings emerged. A prospective clinical observation assessed 50 burn wounds across 30 patients using hydrogel sheets, treating both full-thickness and partial-thickness burns as well as donor areas, and reported no adverse events: this treatment notably reduced pain and enhanced wound healing [62]. Another study evaluated the clinical safety and efficacy of a novel thermos-reversible polyhexanide-preserved wound cover. This randomized, controlled, single-center trial involved 44 patients, comparing a hydrogel containing polyhexanide with an ointment containing sulfadiazine. The results indicated that the hydrogel group experienced less pain and wound staining, underscoring its safety and effectiveness [63]. Additionally, a retrospective observational study on 21 patients using a hydrogel mask for facial burns demonstrated remarkable outcomes, as complete epithelialization occurred in approximately 10.86 days. The hydrogel mask contributed to improved healing and reduced scarring in patients with second-degree facial burns [64]. Together, these studies highlight the promise of hydrogel-based treatments in enhancing burn care and patient recovery. On the other hand, diabetic wounds often lack normal skin regeneration capabilities, making them difficult to heal naturally. These wounds typically appear on the limbs, especially the feet, and are susceptible to infection and recurrence. Severe, full-thickness burns affect all skin structures, including blood vessels and nerves, and can extend to muscles and bones, often leading to hypertrophic scarring [65]. Hydrogel dressings containing stem cells can enhance wound healing by releasing cell growth factors and exosomes while working synergistically with medications (shown in Figure 4) [66].

### 3.2. Delivery of Therapeutic Agents

**Figure 5:**
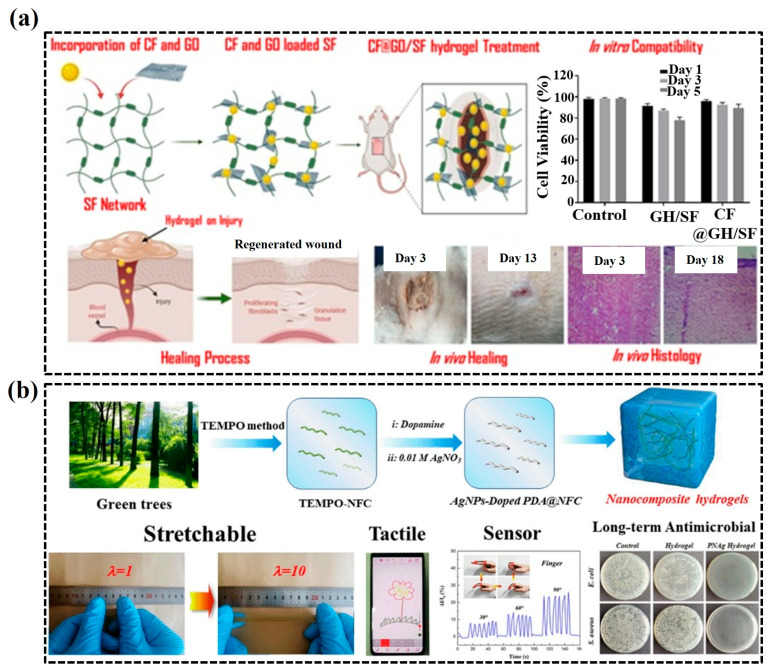
(**a**) The synthesis of graphene/silk cellulose composite hydrogel infused with ciprofloxacin (CIP) and its application in wound healing; reproduced with permission from Ref. [75], Springer Nature. (**b**) Antibacterial hydrogels with metal nanoparticles: fabrication and characterization of cellulose composite hydrogel for sensor applications; reproduced with permission from Ref. [83], Elsevier.

Over the years, antibiotics have served as a cornerstone in the fight against bacterial infections, achieving remarkable success and becoming the treatment of choice [67,68]. However, with the growing challenge of drug-resistant bacteria, the need for innovative solutions is increasingly critical. Traditional systemic antibiotic treatments often lack control, resulting in resistance and side effects [69,70]. In response, researchers have turned to hydrogels—these versatile materials offer a promising alternative by enabling localized antibiotic delivery, thereby reducing misuse and enhancing efficacy [71,72]. One noteworthy advancement is the development of ciprofloxacin-loaded graphene/silk fibroin hydrogels. Ciprofloxacin, a powerful fluoroquinolone, combats a broad range of infections, disrupting bacterial DNA synthesis [73,74]. Zhu et al. demonstrated that these hydrogels effectively inhibit *Pseudomonas aeruginosa* and *S. aureus*, supporting accelerated wound healing, as shown in Figure 5a [75]. Beyond ciprofloxacin, antibiotics like clindamycin have also been integrated into hydrogels. Known for treating severe skin infections caused by *S. aureus*, clindamycin’s efficacy is boosted when combined with hydrogels. Sadeghi et al. crafted a novel antibacterial dressing using clindamycin, carboxymethyl cellulose, and keratin, revealing high cell viability and potential for skin repair [76,77]. Similarly, clindamycin-loaded glycerin hydrogels developed by Jiang et al. show robust antimicrobial activity [78]. In parallel, vancomycin-loaded hydrogels, engineered with oxidized hyaluronic acid, exhibit potent antibacterial effects against gram-positive bacteria, proving especially effective for orthopedic applications [79,80]. Nanotechnology further enhances hydrogel capabilities. Incorporating nanoparticles like silver and copper into hydrogel structures strengthens their physical and antibacterial properties. For example, silver nanoparticle-embedded polyacrylamide networks and copper nanoparticle-based nanocomposites offer robust antibacterial functions and durability [81,82]. Antibacterial hydrogels incorporated with metal nanoparticles are shown in Figure 5b [83].

**Figure 6:**
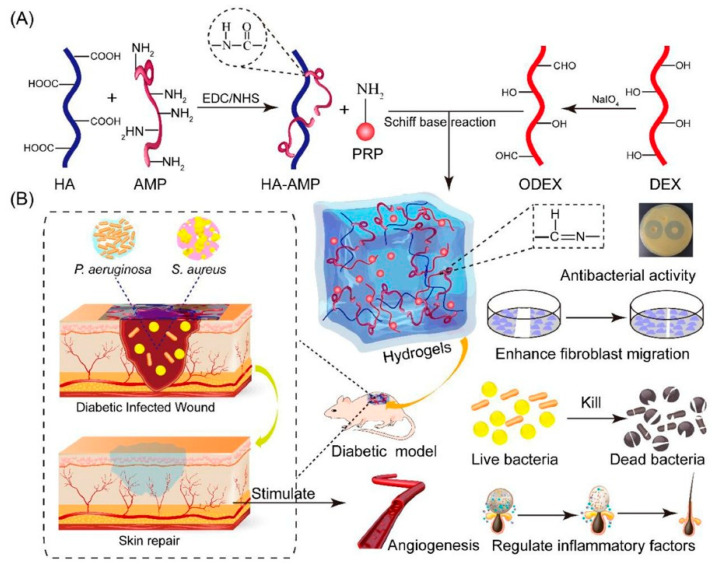
(**A**) Schematic depiction of HA-AMP, OEDX, and multifunctional ODEX/HA-AMP/PRP hydrogel synthesis. (**B**) Schematic overview of composite hydrogels used as wound dressings for healing diabetic infected wounds and skin reconstruction; reproduced with permission from Ref. [85], Elsevier.

Liu et al. developed an antimicrobial peptide (AMP)-infused hydrogel coating using sulfobetaine methacrylate and acrylic acid as monomers [84]. AMPs with two distinct amino acid residues were chemically grafted into the hydrogel. This coating exhibited strong antibacterial activity against both gram-negative and gram-positive bacteria, as well as notable antithrombotic effects. On the other hand, composite hydrogels, using oxidized dextran and platelet-rich plasma, promote wound healing while effectively combating bacteria in diabetic mice. Wei et al. developed a composite hydrogel utilizing Schiff base linkages, which include oxidized dextran, platelet-rich plasma, hyaluronic acid (HA), and a short peptide of 23 amino acids known as cecropin, as depicted in Figure 6 [85].

**Figure 7:**
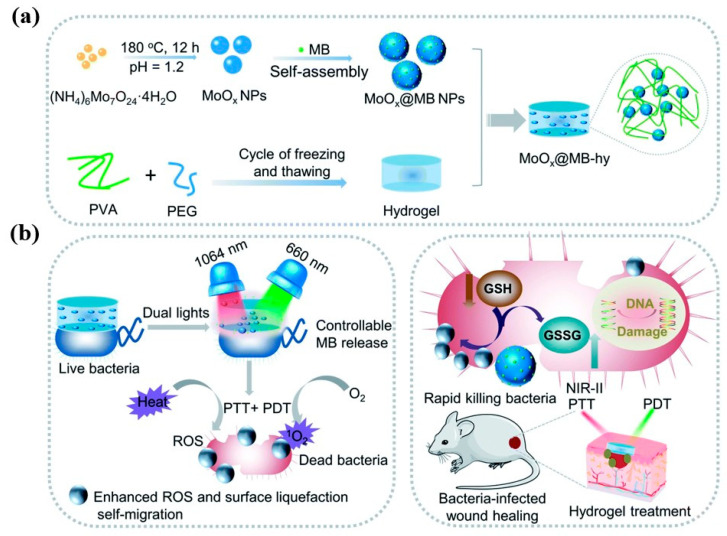
(**a**) Synthesis of photodynamic and photothermal synergistic antibacterial hydrogels (MoOx@MB). (**b**) Schematic illustration of the synergistic antibacterial effect of PTT-PDT [87].

Amidst the challenges posed by drug-resistant infections, combining photodynamic therapy (PDT) and photothermal therapy (PTT) offers a compelling strategy. These techniques synergistically enhance antibacterial efficacy by damaging bacterial membranes and exploiting reactive oxygen species. The creation of a nanocomposite hydrogel with molybdenum oxide nanoparticles and methylene blue by Wang et al. underscores the potential of such approaches in tackling resistant strains, as shown in Figure 7a [86,87]. They also synthesized a hydrogel dressing that can be used for diabetic wound healing. These innovations illustrate a dynamic shift towards using hydrogels and nanotechnology to overcome the limitations of conventional antibiotics, paving the way for more effective and targeted antibacterial therapies within 15 min, as shown in Figure 7b.

Antibiotic resistance continues to be a global health challenge, presenting serious threats worldwide. Bacteriophage (phage) therapy offers a promising alternative to combat antibiotic-resistant infections. Leveraging the properties of hydrogels for delivering biological molecules, as these materials have been formulated to transport phages effectively. Due to their high water content, hydrogels mimic the characteristics of living tissues, allowing them to host proteins, living cells, and biomolecules, thereby extending their biomedical applications [88,89]. Moreover, hydrogels facilitate the controlled release of drugs, benefiting from their adjustable physical properties and biodegradability [90], which is also applicable for delivering biomolecules [91]. These advantageous traits make hydrogels a viable option for phage delivery. Utilizing the combined benefits of phages and hydrogels, phage-loaded hydrogels have been explored for treating and preventing multidrug-resistant (MDR) bacterial infections. Recent preclinical in vitro and in vivo studies increasingly support the potential of hydrogels as an effective delivery system for phages.

Alginate is a natural polymer noted for forming hydrogels that offer excellent biodegradability, biocompatibility, low toxicity, and ease of gelation [92]. It is frequently used in commercial wound dressings, aiding in wound healing while reducing bacterial infections. Additionally, alginate hydrogels can be injected into the body with minimal invasiveness. Similarly, PEG (Polyethylene Glycol) hydrogels crosslinked with a polyurethane matrix have been employed in coating catheters with phages to prevent urinary tract infections [93,94,95]. This usage leverages the hydrophilic and injectable nature of the polyurethane matrix to enhance the delivery and antimicrobial effectiveness of the phages on catheter surfaces. PEG hydrogels integrated with a polyurethane matrix have been utilized to apply phages onto catheters, aiming to prevent urinary tract infections [96]. Phages are covalently bonded to the surface of PEG hydrogels through urethane linkages, connecting the amine groups on the phages to the hydroxyl groups on the hydrogel [97]. This allows the phages to attach efficiently to PEG hydrogel-coated catheters, delivering an antimicrobial effect. The inclusion of a polyurethane matrix in the catheter coating is beneficial because of its hydrophilic nature, smooth texture, and ease of injection [98]. Systemic antibiotics used for bone infections often struggle with poor site-specific delivery and can lead to nephrotoxic and hepatotoxic effects at high doses [99]. To address this challenge, injectable hydrogels have been investigated for their ability to locally deliver phages that kill multidrug-resistant (MDR) bacteria associated with orthopedic implant infections. For instance, Wroe et al. demonstrated that a PEG-4-MAL hydrogel encapsulating Paer14 phages led to a 17-fold reduction in *P. aeruginosa* biofilm formation compared to control gels. The phage-treated hydrogels had significantly more dead bacteria, as confirmed by fluorescence staining. Similarly, Barros et al. observed that phages like LM99 formulated in alginate hydrogels dramatically reduced planktonic MDR Enterococcus faecalis cells by 99% and bacterial attachment by 98% within 24 h in vitro [100]. In healthcare facilities, catheters are commonly used but are vulnerable to biofilm formation, leading to catheter-associated urinary tract infections (CAUTIs) [101]. To combat such infections, which are often due to MDR pathogens, phage-loaded hydrogels are emerging as a strategic solution to prevent and disrupt biofilms. Studies have shown that hydrogels incorporating phages active against bacteria such as *S. epidermidis*, *P. aeruginosa*, *E. coli*, and *P. mirabilis* can significantly diminish biofilm accumulation on catheter surfaces [102,103]. In addition, burn injuries, which are among the top four most severe forms of trauma globally, create environments conducive to bacterial growth and can result in fatal outcomes if untreated [104,105]. Given their high local bioavailability, topical antimicrobial treatments are often preferred over systemic antibiotics [106]. Hydrogels play a vital role in wound care by maintaining a moist environment that promotes healing and the absorption of exudates and facilitates self-repair [107]. Phages can be incorporated into these hydrogels to treat bacterial wound infections effectively.

#### 3.2.1. Antibacterial Application

**Figure 8:**
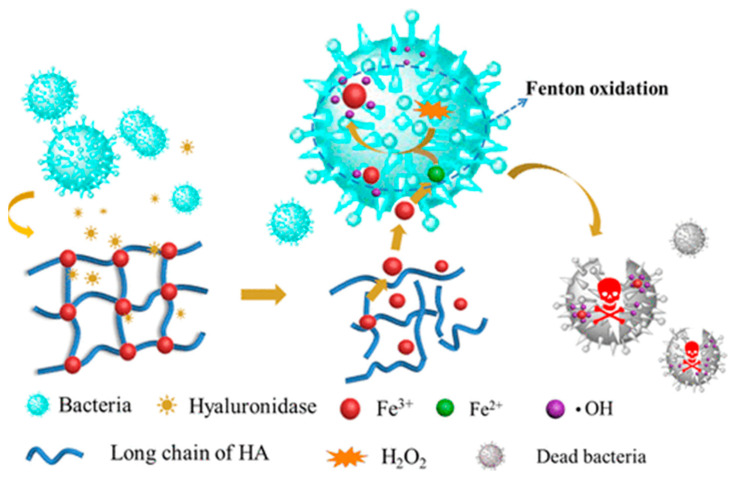
Mechanism of bacteria-induced antimicrobial activation in HA−Fe−EDTA hydrogel [108].

In developing antibacterial hydrogels, methods include embedding antibacterial agents, like antibiotics and metal nanoparticles, or integrating antibacterial components through physical or chemical crosslinking. A hydrogel created by combining hyaluronic acid with a chelating agent complex featured self-healing and antibacterial functions. In this system, the metal ions act as both a crosslinking agent and a nontoxic antibacterial agent. The hydrogel disintegrates in the presence of bacterial hyaluronidase, releasing Fe^3+^ complexes. These complexes, once absorbed by the bacteria, are reduced to Fe^2+^ by H_2_O_2_, leading to the production of hydroxyl radicals that disrupt bacterial proteins and nucleic acids, ensuring sustained antibacterial efficacy (shown in Figure 8) [108].

**Figure 9:**
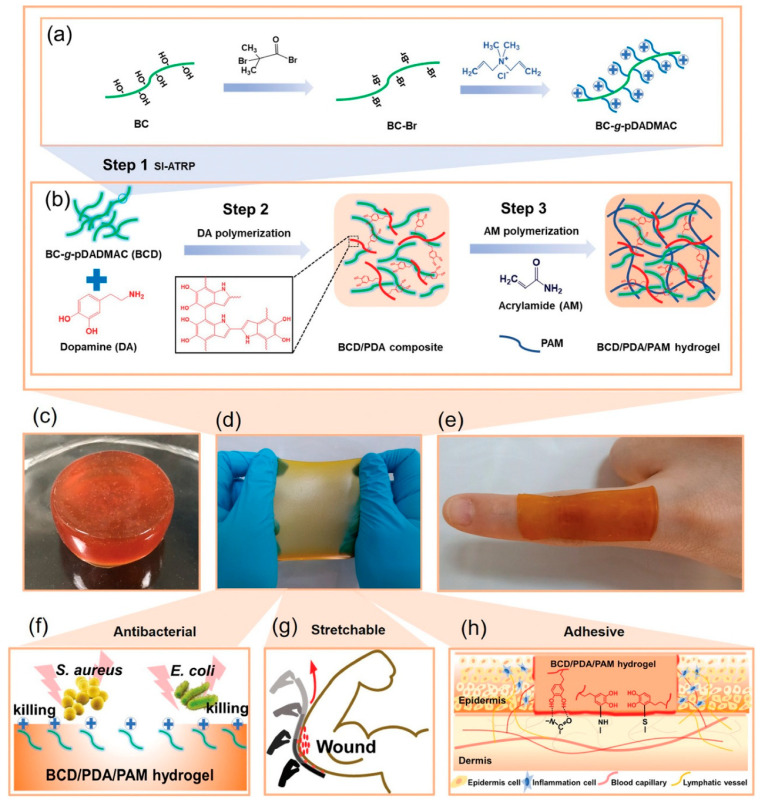
Schematic of the stretchable, adhesive, and antibacterial hydrogel. (**a**) Preparation of bacterial cellulose derivative by using surface-initiated atom transfer radical polymerization graft from bacterial cellulose. (**b**) Formation of hydrogel. (**c**–**e**) Different shapes of hydrogel. (**f**) Quaternary ammonium salts of bacterial cellulose derivative provide the antibacterial hydrogel. (**g**) Stretchable hydrogel covers the elbow joint completely. (**h**) Catechol groups of polydopamine components promote tissue adhesion of hydrogel [112].

To mitigate the risk of wound infections, contemporary research has focused on the incorporation of functional agents such as metal ions (e.g., zinc and silver), antimicrobial peptides (AMPs), and various bioactive compounds into hydrogels. These enhancements have proven effective in significantly improving the antibacterial properties of these materials. Numerous studies have explored the use of various inorganic metal ions, including silver, copper, zinc, and gold ions, as well as metal oxides, like copper oxide and zinc oxide, and their respective nanoparticles, as broad-spectrum antibacterial agents suitable for incorporation into hydrogels [109]. Among the different metal agents, silver ions are widely recognized as a quintessential antibacterial component, with proven success in clinical applications. The antibacterial mechanism of silver ions involves their binding to the negatively charged thiol groups present in bacterial membrane proteins, leading to protein denaturation and, ultimately, bacterial cell death [110]. The focus of recent investigations has also highlighted the incorporation of metal nanoparticles, such as silver, copper, magnesium, and zinc oxides, into hydrogels. Rastegari et al. [111] characterized a chitosan-based hydrogel embedded with zinc oxide nanoparticles (ZnO NPs) to facilitate the controlled release of vancomycin, enhancing its antibacterial efficacy. While the inclusion of ZnO NPs led to a reduction in the overall release rate of vancomycin, the issue of burst release persisted—approximately 30% of the drug was released within the first two hours. This phenomenon raises concerns regarding biocompatibility. Nonetheless, the resultant hydrogels exhibited high antibacterial efficiency against both *Staphylococcus aureus* and *Pseudomonas aeruginosa*, indicating promising potential for clinical applications. There are complexities associated with wound treatment in high-movement areas; traditional cotton gauze presents several limitations, such as inadequate surface coverage, restricted joint movement, lack of antibacterial functionality, and the necessity for frequent replacements. Hydrogels are increasingly explored as viable alternatives due to their inherent flexibility and biocompatibility. However, conventional hydrogels often fall short in terms of adhesion, mechanical strength, and antibacterial properties. Yang Zifeng et al. [112] addressed recent innovations that incorporate cationic polyelectrolyte brushes grafted from bacterial cellulose (BC) nanofibers into polydopamine/polyacrylamide hydrogels. These polymer brushes, with their rigid BC backbones, significantly enhance the mechanical properties of the hydrogels, achieving impressive tensile strength and strain resistance. The incorporation of quaternary ammonium groups imparts lasting antibacterial effects and aids in promoting the crawling and proliferation of epidermal cells. Furthermore, the catechol groups within the hydrogels provide robust adherence capabilities, ensuring stability even in areas subjected to extensive movement (shown in Figure 9).

**Figure 10:**
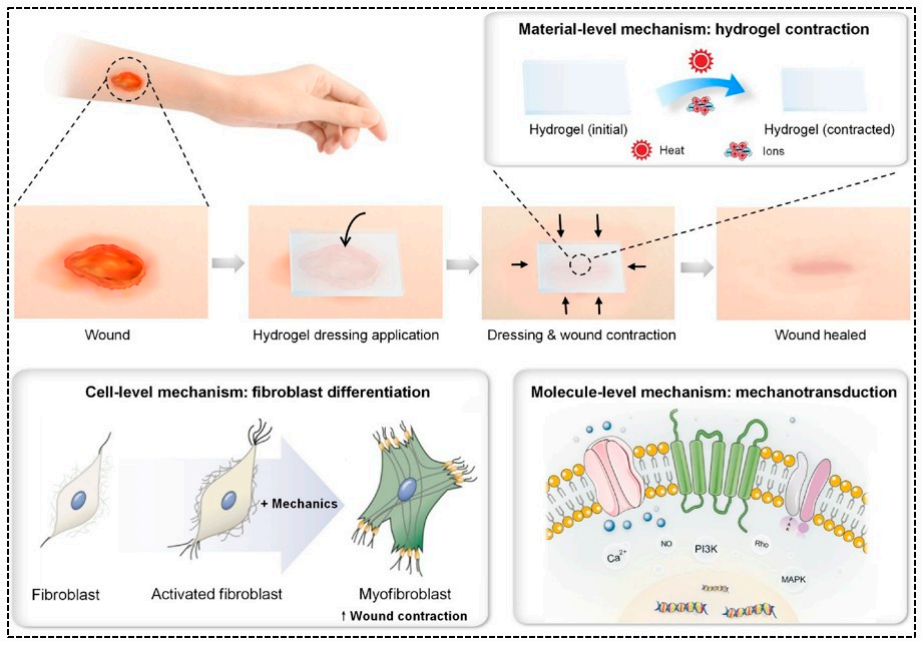
Mechanically active wound healing hydrogels: stimuli-responsive dressings contract to enhance healing, inducing fibroblast differentiation into myofibroblasts and activating gene expression changes via the PI3K pathway; reproduced with permission from Ref. [123], Elsevier.

Intelligent hydrogels with antimicrobial properties have been developed to combat infections and promote wound healing [113,114]. These smart hydrogels function as drug delivery systems, releasing drugs in controlled amounts in response to environmental triggers, and have been designed as wound dressings that gradually release antibacterial agents, thus preventing the colonization of pathogens at the wound site [48,49]. A variety of pH-sensitive hydrogels have been created for these purposes, with their swelling behavior adjusting to changes in environmental pH [115]. This property has been particularly useful in fabricating wound dressings that inhibit infections and aid in healing. For instance, antimicrobial hydrogels incorporating pH-responsive keratin/zinc oxide nanoparticles (nZnO) have shown tunable properties, with the ZnO release decreasing more at an acidic pH of 4 compared to a basic pH [116]. These hydrogels demonstrated antibacterial efficacy against *S. aureus* and *E. coli* using Japanese industrial standards and disk diffusion methods. Another innovation by Khan and colleagues involved blended hydrogel films with pH sensitivity for wound dressing applications, exhibiting substantial antibacterial activity [117]. Meanwhile, a silver nanocomposite hydrogel developed by Sudarsan et al. showcased its antimicrobial prowess, effective against *E. coli* and *S. aureus*, via the well diffusion method [118]. The incorporation of thermo-responsive features into hydrogels has also been explored. Mi et al. introduced a multifunctional antimicrobial wound dressing hydrogel using ABA triblock copolymers with segments of PNIPAM and PCBAA-1-C2 SA. This hydrogel demonstrated controlled drug release capabilities, reducing infection risks and expediting the healing process [119]. Zhao et al. developed a thermo-responsive chitosan/β-glycerophosphate hydrogel with Ag NPs decorated on reduced GO nanosheets, significantly improving the healing of MRSA-infected wounds both in vitro and in vivo [120]. A chitosan-based thermo-sensitive hydrogel was also crafted for treating extensively drug-resistant A. baumannii-infected wounds, enhancing therapeutic outcomes. Furthermore, some synthetic hydrogels are engineered to respond to light stimuli. Wang et al. created a cross-linkable, blue-light-sensitive hydrogel that combines two different chitosan chains and demonstrated nearly 100% antibacterial activity against *E. coli* and *S. aureus*. This hydrogel also promoted hemostasis and combated wound infections effectively in vivo. Enzyme-sensitive hydrogels are another layer of innovation, using enzymes as signals for targeted drug delivery. These hydrogels respond to specific enzymes, like glycosidases and proteases, offering enhanced specificity and selectivity [121]. Zuo et al. developed an enzyme-responsive antibacterial hydrogel with Ag NPs, showcasing excellent performance against *S. aureus* in both laboratory and live models [122]. The applications of these advanced hydrogels highlight their potential to revolutionize wound care and infection control by leveraging their stimulus-responsive features to optimize antimicrobial delivery and efficacy. Le Chang and colleagues explore the use of mechanically active dressings (MADs) that utilize self-contractile hydrogels to aid in wound contraction. These dressings have demonstrated enhanced healing rates with minimal side effects, highlighting a significant advancement in wound care technology. Recent studies have focused on stimuli-responsive hydrogels for creating MADs due to their strong adhesion to the moist and curved surfaces of biological tissues, as well as their ability to provide controlled contraction in response to various external stimuli, effectively pulling the wound edges inward to promote closure (shown in Figure 10) [123,124,125].

#### 3.2.2. Anti-Inflammatory Action

Hydrogels have been tailored to deliver specific anti-inflammatory drugs, such as dexamethasone or ibuprofen, directly to inflamed tissues. These hydrogels can be designed with a particular chemical composition that allows them to swell or release their drug load in response to inflammatory triggers like increased temperature or changes in pH that occur at the site of inflammation. For example, a hydrogel loaded with dexamethasone could release the drug slowly over time, maintaining a therapeutic level for an extended period, which improves the drug’s efficacy while minimizing the frequency of dosing and potential side effects. Additionally, such hydrogels can be applied topically or injected directly into the affected area, ensuring that the drug acts locally rather than systemically, further reducing the possibility of systemic side effects. Diclofenac is recognized as a highly effective and widely prescribed non-steroidal anti-inflammatory drug (NSAID), but its use has been associated with increased coronary and vascular risks, as well as gastrointestinal complications [126]. Efforts to develop safer delivery methods have led to the formulation of lipid-based gels for transdermal delivery of diclofenac. A study by [127,128] reports the preparation of a microemulsion-based hydrogel by incorporating diclofenac into the oily phase of a microemulsion using limonene and then gelling it with Carbomer-940. This method aims to deliver therapeutically sufficient amounts of diclofenac while minimizing systemic side effects. A new polymer hydrogel platform is designed to release anti-inflammatory molecules in response to human blood’s inflammatory triggers. In this system, anti-inflammatory peptides targeting the complement system or cyclophilin A are linked to the hydrogel using peptide sequences that elastase, released by activated granulocytes, can cleave. This adaptive drug delivery is initiated by activated granulocytes, demonstrating an effect on respective inflammatory pathways. The drug release is tunable to micromolar doses by adjusting the gel’s functionalization degree. In a related approach, anticoagulant hydrogels were developed to release heparin based on coagulation status [129]. These are composed of starPEG and heparin, using peptide linkers cleaved by coagulation factors to release heparin, stopping further hydrogel cleavage. Elastase, active during inflammation, serves as a trigger in these systems. Aimetti et al. recently highlighted neutrophil elastase-cleavable hydrogels for effective drug release [130]. Additionally, elastase-responsive nanoparticles were designed for lung inflammation [131]. All these systems embed bioactive molecules within the hydrogel network, releasing them through elastase-triggered proteolytic cleavage.

### 3.3. Hydrogel for Cancer Treatment

**Figure 11:**
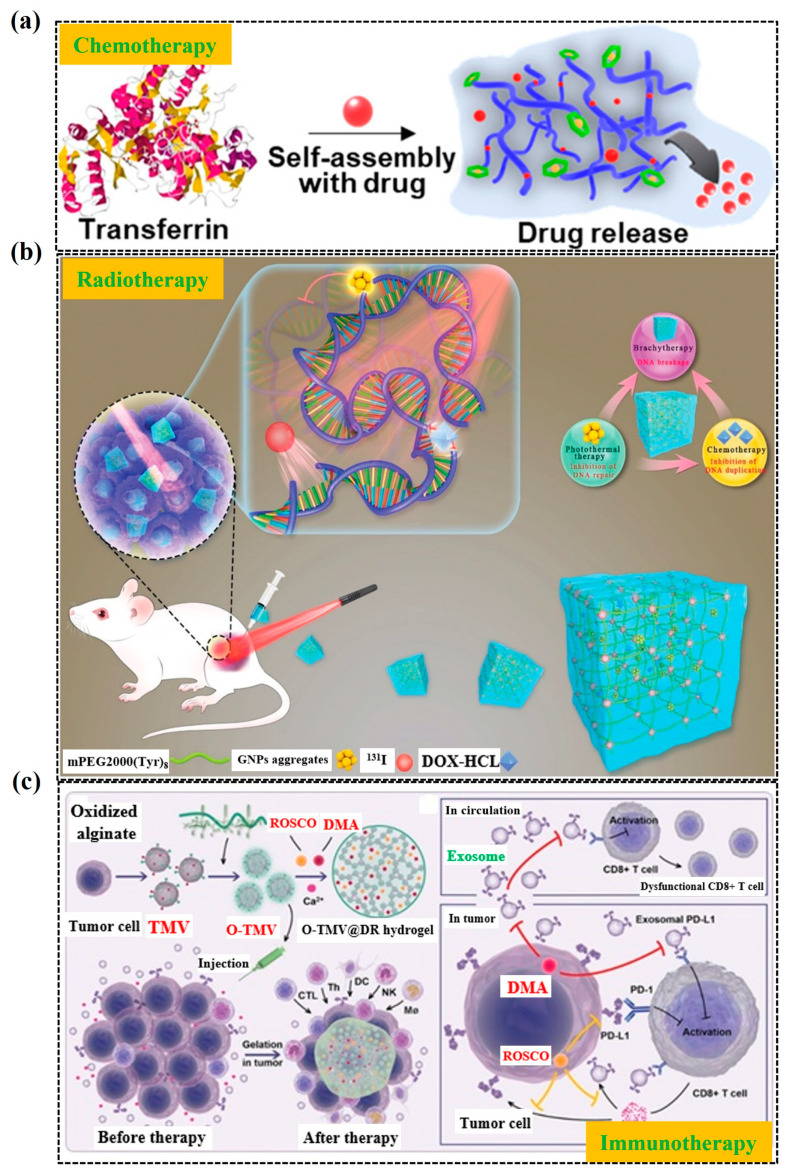
(**a**) Self-assembly with drug release with hydrogel with chemotherapy; reprinted with permission from [134]. Copyright © 2021, American Chemical Society. (**b**) The potential mechanisms involved in the triple-combination synergistic antitumor with therapy; reproduced with permission from [142]. Copyright © 2021, Wiley and Sons. (**c**) An injectable hydrogel with membrane vesicles, DMA, and ROSCO targeting tumor before and after therapy. Reproduced with permission from [143]. Copyright © 2021, Wiley and Sons.

Hydrogels, with their biocompatible and biodegradable properties, are revolutionizing targeted cancer therapy by enabling precise and controlled drug delivery. These smart materials can be engineered to respond to specific tumor characteristics like pH and temperature, ensuring the release of therapeutic agents directly at the cancer site, which minimizes damage to healthy tissues and reduces systemic toxicity. By incorporating targeting ligands, hydrogels enhance the precision of drug delivery, making treatments more effective while lowering side effects. Additionally, they offer the capability to co-deliver multiple agents, such as drugs and nanoparticles, in a single system, providing a comprehensive approach to cancer treatment. Their use supports minimally invasive administration, making them a promising tool in enhancing the efficacy and safety of cancer therapies. Hydrogels are characterized by their superior biocompatibility, biodegradability, and capacity for drug loading and controlled release, making them a popular choice in various cancer treatments. They are extensively utilized in modalities such as radiotherapy, chemotherapy, and immunotherapy (shown in Figure 11). Hydrogels, renowned for their superior biocompatibility and biodegradability, are paving the way as cutting-edge carriers for cancer drugs, offering several remarkable benefits. To start, they function as sophisticated systems for the precise, controlled release of therapeutic agents, such as chemotherapeutics, radionuclides, immunosuppressants, and more. This capability is leveraged across various treatment modalities, like chemotherapy, radiotherapy, immunotherapy, hyperthermia, and photo-based therapies, ensuring a comprehensive approach to cancer treatment. Additionally, hydrogels can be customized in terms of size and delivery path, allowing them to precisely target diverse cancer types and locations, which enhances drug specificity, minimizes dosage requirements, and increases treatment success. Moreover, their ability to respond to internal and external environmental stimuli enables the smart, remote-controlled release of anticancer agents. Collectively, these attributes are transforming hydrogels into pivotal tools in cancer care, offering new hope for improving patient survival outcomes and enhancing quality of life.

Kim et al. [132] designed a hydrogel-based drug delivery system that simultaneously releases doxorubicin (DOX) and 5-fluorouracil (Fu) over an 18-day period. This system involved Fu-loaded Pluronic and diblock copolymer hydrogels (Fu-HP and Fu-HC) combined with DOX-loaded microcapsules (DOX-M), creating two effective drug depots. Both formulations allowed for easy injection into tumors and gelled in situ at body temperature. This microcapsule–hydrogel system offered a prolonged, controlled release of drugs, reducing sudden spikes in concentration and minimizing toxicity compared to standard microcapsules. Chemotherapy plays a significant role in cancer treatment, often serving as an adjunct to surgical interventions or in combination with other therapies to eliminate cancer cells. However, conventional chemotherapeutic agents frequently face issues, such as severe side effects, low drug tolerance, and suboptimal targeting. As a result, while chemotherapy may not eradicate all cancer cells, it can inflict considerable harm on the body [133]. This is where hydrogels emerge as promising new drug carriers, as they excel in controlling drug loading and release, facilitating effective cancer drug delivery with long-lasting effects. For instance, Lee et al. [134] developed a temperature- and pH-responsive self-assembled hydrogel that incorporates anticancer drugs mixed with a saline solution containing transferrin and dithiothreitol. By adjusting the temperature and pH of the solvent, the release rate and quantity of the anticancer drugs from the hydrogel can be finely controlled. This effective, sustained release resulted in an 80% reduction in cancer cells after 48 h of incubation, demonstrating a high efficacy in inhibiting cancer. The simple formation process of self-assembled hydrogels that made it easier to package the desired number of anticancer drugs is shown in Figure 11a. In parallel, radiation therapy—often employing high doses (over 60 Gy) to eradicate tumor cells and reduce cancer size—has become a standard and effective method in cancer treatment [135]. The focus of radiation therapy has shifted from broad-spectrum to highly targeted approaches that efficiently destroy cancer cells while minimizing damage to surrounding healthy tissues by disrupting the tumor cells’ DNA repair mechanisms [136]. However, the effectiveness of radiotherapy can be compromised by the inherent DNA repair capabilities of cancer cells, their radiation resistance under hypoxic conditions, and uneven radiation dose distribution from localized administration. Consequently, while radiotherapy is typically more effective for early-stage cancers, its efficacy in intermediate to late-stage or metastatic cancers remains uncertain [137,138]. In this context, hydrogels featuring complex, multi-network, and porous structures demonstrate advantageous properties, including adaptability and responsiveness to various stimuli. They can evenly distribute precise doses of radionuclides throughout tumor cells [139] and simultaneously carry radiosensitizers, chemotherapeutic agents, photosensitizers, and heat-sensitive compounds. This capability enables hydrogel systems to inhibit DNA self-repair mechanisms and reduce hypoxia-induced radiation resistance in cancer cells, resulting in compounded damage to DNA and enhancing the effectiveness of brachy therapy treatments [140]. For instance, Wang et al. [141] introduced an injectable hydrogel composed of Endostatin (ES) and hyaluronic acid–tyramine, which effectively sustains the release of ES. This sustained release considerably decreases cancer microvascular density and hypoxia, thereby significantly improving the sensitivity of cancer cells to radiation therapy. Thus, the integration of hydrogels in both chemotherapy and radiotherapy not only addresses the limitations of conventional treatments but also offers innovative approaches to enhance cancer treatment outcomes. J. Zhang et al. have developed a highly efficient platform for synergistic tumor therapy, providing insights into antitumor mechanisms at the DNA level. Figure 11b [142] schematically illustrates the potential mechanisms of the triple-combination synergistic antitumor therapy. Furthermore, the innovative use of an injectable hydrogel creates an immune checkpoint-regulatable niche for cancer immunotherapy, as shown in Figure 11c [143].

To effectively address tumor heterogeneity, it is essential to use a combination of treatment modalities, such as chemotherapy and radiotherapy. Hydrogels present a promising platform for the co-delivery of drugs in cancer chemoradiotherapy and are typically classified into two types: those that require external radiation (e.g., X-ray or gamma radiation) to trigger polymerization or drug release at tumor sites and those that contain encapsulated radioisotopes (such as iodine-131, copper-67, or rhenium-188) for internal radiotherapy [144,145]. For instance, a study utilized doxorubicin (DOX) and iodine-131-labeled hyaluronic acid (131I-HA) within a hydrogel framework, which demonstrated significant antitumor effects both in vitro and in vivo. After subcutaneous injection, the 131I isotope and DOX were maintained at the tumor site for approximately four weeks with minimal organ damage. This hydrogel setup provides precise control over drug release in a single administration, facilitating the simultaneous delivery of multiple therapeutic agents and highlighting the spatiotemporal advantages of hydrogels for localized treatment. Additionally, integrating chemotherapy with gene therapy to combat tumor angiogenesis and resistance to chemotherapeutics necessitates the use of therapeutic genes, and positively charged hydrogels can form stable complexes with negatively charged nucleic acids for effective delivery. Liu et al. proposed a hydrogel for peritumoral injection composed of α-cyclodextrin (α-CD) and a positively charged amphiphilic copolymer featuring a folic acid-targeting moiety [146,147,148]. Yang et al. extensively explore hydrogel-based delivery systems, focusing on their potential for the localized treatment of brain tumors by smart hydrogels capable of bypassing the blood–brain barrier and infiltrating larger tumor regions, enabling precise and controlled drug release. Hydrogel-based drug delivery systems are presented as a transformative solution to overcome the limitations of conventional brain cancer therapies [149].

## 4. Microgels

In the exciting field of materials science, researchers have been exploring a fascinating class of materials known as microgels. These soft, deformable, and permeable polymeric particles are emerging as multifunctional solutions for an array of applications, including drug delivery, interface stabilization, cell cultures, and innovative approaches to tissue engineering. Among the various types of microgels, cellulose microgels are particularly noteworthy due to their biopolymeric characteristics, which offer distinct advantages. Sporting an abundant hydroxyl structure, these microgels can be meticulously designed and feature a complex multiscale pore network that enhances their compatibility with biological systems, making them particularly suited for biomedicine. Microgels can be characterized as highly swollen colloidal entities formed from cross-linked polymers that create a robust internal gel-like network. Their synthesis can follow two main avenues: the bottom-up approach, which involves the controlled growth of polymer networks, or the top-down method, where larger hydrogels are broken down into microscale particles [150,151]. This flexibility in production not only widens their applications but also permits customization for specific functions. One of the standout attributes of microgels is their unique properties, which set them apart from other types of particles. Their microscale polymeric network structure and extensive surface area endow them with exceptional permeability, allowing for effective absorption and release of materials. They can also respond dynamically to external changes, swelling or shrinking in a reversible manner, thus making them excellent carriers for active substances such as drugs, bioactive molecules, and biochemical signals. Recent research has introduced multiresponsive supramolecular PNIPAM microgels utilizing a metallo-supramolecular cross-linker instead of the conventional BIS. This cross-linker adds salt responsiveness and degradability to the microgels’ thermoresponsive properties. The use of sodium dodecyl sulfate (SDS) in synthesis allows for adjustable microgel size and the incorporation of hydrophilic, positively charged cross-linkers. These microgels exhibit a core shell-like structure and hold promise for applications in stabilizing smart emulsions and drug delivery systems, highlighting advancements in stimuli-responsive materials [152,153].

Moreover, microgels exhibit remarkable interfacial behavior. They strongly adhere to oil–water or air–water interfaces due to their surface-active characteristics, significantly reducing interfacial tension. These highly swollen polymer networks mimic the composition and mechanical properties of the extracellular matrix found in living organisms, which contributes to their outstanding biocompatibility and degradability [154]. This unique trait facilitates not only the controlled release of encapsulated substances but also enables their use in in vitro cell culture and in vivo cell transplantation [155]. The controlled pore structures and expansive internal surfaces of microgels provide ample adhesion sites for cells, promoting nutrient transport through their porous network. Consequently, individual microgel particles can be employed as building blocks in tissue engineering, allowing for the construction of highly regulated multifunctional platforms aimed at tissue repair, regeneration, and the creation of advanced 3D printed scaffolds. As research advances, scientists are increasingly drawn to natural, bio-based microgels, praised for their excellent biocompatibility and degradability [156,157,158,159]. These materials hold significant promises for future biomedical applications, enhancing drug delivery systems and pioneering new approaches to health care. Through ongoing studies, microgels are poised to revolutionize material applications in medicine and beyond, offering innovative solutions that can benefit society as a whole.

### Fabrication of Microgels

**Scheme 3:**
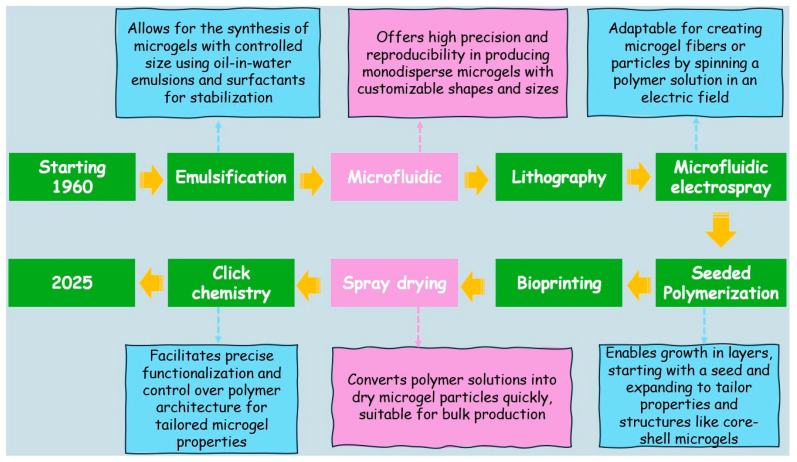
Distinctive features and characteristics of strategies used in microgel fabrication.

Microgels are fascinating materials that can be synthesized from natural or synthetic polymers through both covalent and non-covalent cross-linking techniques [160,161]. Based on the nature of cross-linking, microgels are grouped into two primary categories: chemical microgels and physical microgels. Chemical microgels are constructed using covalent bonds, typically facilitated by cross-linking agents or enzymes such as glutaraldehyde, epichlorohydrin, and glutamine aminotransferase, creating robust polymer networks [162,163]. In contrast, physical microgels form their networks through non-covalent interactions—such as hydrogen bonds, hydrophobic interactions, and ionic or electrostatic forces—under specific environmental conditions like temperature, pH, or ionic strength. Although these physical interactions provide a degree of flexibility, they tend to be reversible and less stable than covalent bonds [164,165]. With the growing interest in microgels over the past twenty years, numerous fabrication techniques have been developed and refined. These methods can be categorized into four fundamental groups based on their physical principles: micro-molding, emulsion techniques, shearing methods, and extrusion processes [166]. Each of these fabrication strategies involves distinct requirements for materials and equipment. For instance, micro-molding necessitates specialized molds, while emulsion methods leverage a combination of immiscible liquids—primarily oil and water—mixed in a way that creates water-in-oil droplets, eliminating the need for a mold. The details of the fabrication strategy are shown in Scheme 3. In the “top-down” approach to microgel creation, larger macro gel structures are mechanically broken down, a process often referred to as broken gels. This can be accomplished through various mechanical means, including grinding, ultrasound, or high-pressure homogenization. The characteristics of the microgels produced by top-down methods, including size and shape, are influenced by factors such as the type of raw material used, the applied stress levels, and processing rates and durations [167,168].

Alternatively, precision in microgel geometry can be achieved using designed masks or molds in the top-down approach. Lithography techniques—such as imprint lithography, photolithography, or flow lithography—employ specific patterns to dictate the size and shape of the resulting microgels. The lithography process generally involves creating a mold that is formed through a multi-step process, most often employing silicon due to its durability and thermal stability [169,170]. Photolithography is a distinctive method within lithography that utilizes light to pattern photosensitive polymers, eliminating the requirement for physical molds. In instances where gelation can be induced through UV light, photolithography proves highly effective for microgel fabrication. For example, researchers have successfully employed treated polydimethylsiloxane (PDMS) photomasks to create cell-laden microgels that are highly reproducible in their dimensions [171]. These microgels, measuring 90 µm in width and ranging from 180 to 270 µm in length, were used for the single-fluorescence detection of DNA oligomers when integrated with appropriate probes [172]. Mechanical fragmentation offers another top-down strategy where preformed hydrogels are disintegrated into microgels through applied mechanical force. A common approach involves using a standard kitchen blender to reduce solid gelatin hydrogels into microgel form. The size of the resulting microgels can be controlled by adjusting the blending duration; for example, an average diameter of 55 µm was achieved after blending for 120 s [173]. On the other hand, the “bottom-up” approach directly assembles microgels from individual monomers or polymers, allowing for a high degree of control over the formation of these particles. This method encompasses various mechanical manufacturing techniques, including the emulsion template method, extrusion, and injection, where droplets are created and subsequently cross-linked under optimal conditions—such as through enzyme action, UV light exposure, or thermal treatment—to yield gel particles. By integrating various production mechanisms and synthesis methods, this approach facilitates the generation of diverse and precise microgels tailored for specific biomedical uses [174]. Through these innovative fabrication strategies, the microgel research landscape continues to evolve, presenting promising potentials for applications in drug delivery, tissue engineering, and other cutting-edge fields.

## 5. Application of Microgels

### 5.1. Delivery of Therapeutic Agents/Drug Delivery

Drug delivery systems enhance the efficacy and bioavailability of therapeutic agents through targeted administration. Conventional methods, such as oral and injectable routes, face limitations like poor solubility and systemic side effects. Advanced systems, including targeted drug delivery and nanoparticle-based systems, improve drug delivery and reduce toxicity. Smart systems, like pH-sensitive microgels, enable the controlled release of insulin in response to gastric conditions. Microgels show promise for DNA delivery due to their ability to encapsulate and protect DNA, enhancing cellular uptake by surface functionalization and enhancing cell targeting and transfection efficiency. However, challenges remain in optimizing microgel properties and ensuring biocompatibility. Sunghyun Moon et al. studied the development of injectable, biocompatible systems for controlled immune modulation represents a significant advance in therapeutic strategies with unique properties of dendritic cells (DCs) and the versatility of alginate hydrogels. The encapsulation of microparticles within readily injectable alginate balls achieves consistent room-temperature crosslinking. In vitro data highlight the ability of encapsulated DCs to proliferate for up to 7 days, supporting the potential for sustained immune activation [175]. Carboxymethyl chitosan (CMC) microgels are attractive candidates for drug delivery systems owing to their inherent biocompatibility and biodegradability, coupled with the potential for targeted and controlled release. The introduction of carboxyl groups enhances solubility and provides versatile functionalization sites for ligand conjugation to enable targeted delivery. The porous structure of CMC microgels facilitates controlled release kinetics, often tunable through modification of parameters such as crosslinking density and particle size, with the potential for pH-responsive release in relevant physiological environments [176]. Mehtap Sahiner et al. studied carboxymethyl chitosan microgels, synthesized via microemulsion polymerization, which exhibited tailored size and zeta potential, confirmed by SEM. Biocompatible carboxymethyl chitosan microgels effectively loaded vancomycin, displaying sustained antibacterial activity against *E. coli* and *S. aureus*, particularly against *S. aureus*, for up to 72 h [177]. One study focused on the properties of diverse synthetic (e.g., poly(N-isopropylacrylamide), PEGDA, poly (vinyl alcohol)) and natural (e.g., chitosan, alginate, agarose) hydrogel precursors, emphasizing their biocompatibility and tunability for specific applications. Applications in anticancer drug delivery, immunotherapy, drug screening (particularly within 3D organoid models), and overcoming the blood–brain barrier are reviewed, showcasing the therapeutic potential of these systems [178,179]. It also explored the development of a novel targeted drug delivery system using functionalized poly(N-isopropylacrylamide) (pNIPAM) microgels, which conjugate these microgels with folic acid (FA), exploiting its high affinity for folate receptors often overexpressed on cancer cells, to achieve targeted delivery. Doxorubicin (Dox), a potent chemotherapeutic agent, is then incorporated into the microgel structure for tumor-targeting drug delivery [180].

### 5.2. Cancer Treatment

**Figure 12:**
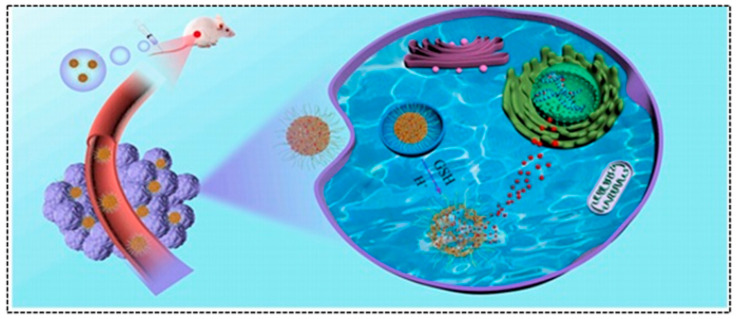
Action of pH-redox responsive degradable and non-degradable microgels: drug release behavior and enhanced anticancer efficacy; reproduced with permission from Ref. [185], Elsevier.

Cancer remains a major global health challenge, claiming millions of lives each year. According to the World Health Organization, cancer was responsible for ten million deaths worldwide in 2020 [181]. A primary strategy in treating cancer has long been chemotherapy, though its non-targeted systemic delivery often results in significant collateral damage to healthy cells [182]. This can lead to a host of adverse side effects, including suppression of bone marrow function, nausea, and, in severe instances, liver and heart complications due to the induced apoptosis not only of cancer cells but also of healthy ones [183]. In recent years, there has been a surge in the development of engineered nano- and micro-scale systems designed for targeted drug delivery. These advancements aim to refine the precision of chemotherapy to specifically attack cancer cells while sparing healthy tissue and thereby minimizing side effects [184]. Scientists’ innovations in creating smart particles capable of homing in on cancer cells mark a promising leap forward in reducing the harsh impact of traditional chemotherapy treatments. Hydrogels play a crucial role in cancer treatment by enabling targeted and controlled drug delivery to tumors, minimizing side effects and enhancing therapeutic efficacy. Di Huang et al. developed a novel drug delivery system aimed at improving breast cancer treatment, utilizing pH- and redox-responsive polymeric microgels. These microgels, crafted through emulsion polymerization, incorporated Doxorubicin (DOX) as the model drug. Key components included DSDMA, serving as a redox-responsive cross-linker, DPA as the monomer, and PEG, which provided hydrophilic shells imparting stability and anti-fouling properties. The microgels exhibited dual responsiveness due to DPA’s pH sensitivity and DSDMA’s disulfide bonds. MTT assays confirmed low cytotoxicity at concentrations of up to 100 μg/mL. Moreover, DOX-loaded microgels effectively inhibited 4T1 cell proliferation and significantly enhanced tumor suppression in vivo, as shown in Figure 12 [185]. Earlier, it was demonstrated that core PNIPAM and core/shell PNIPAM-co-PAA microgels enhance the encapsulation and delivery of methylene blue, with core PNIPAM releasing the drug more slowly, providing sustained therapeutic effects. In vitro studies have shown effective inhibition of MCF-7 cell proliferation, indicating the potential of these microgels as platforms for optimizing anticancer drug delivery [186].

Microgels in ovarian cancer therapy enable precise drug delivery by releasing agents in response to tumor-specific conditions. Coupled with photothermal nanoparticles, they enhance treatment precision and efficacy, offering improved biocompatibility and tumor retention for better therapeutic outcomes. Xiaodong Ma et al. developed a microfluidic technique to fabricate biomimetic nano@microgels, comprising GelMA microspheres containing CAT and Au@MSN-Ter/THPP@CM nanoparticles. These nanoparticles demonstrated remarkable photothermal conversion and stability, while the nano@microgels showed excellent biocompatibility and prolonged tumor retention. Both in vitro and in vivo studies confirmed their potent anti-tumor effects against ovarian cancer with the application of 650 nm and 980 nm lasers, highlighting their potential as an innovative treatment option for ovarian cancer [187].

### 5.3. Microgels-Based Wound Dressings

**Figure 13:**
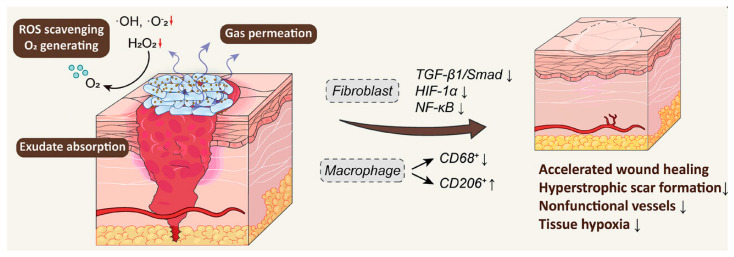
Illustration of the rod−shaped microgel scaffolds for accelerating wound healing and reducing hypertrophic scar formation [190].

In the complex process of skin wound healing and scar formation, the surrounding environment plays a crucial role, with low oxygen levels and inflammation often interacting to create conditions that hinder proper healing [188]. Traditional hydrogel dressings are effective at keeping wounds moist and can have properties that reduce inflammation and oxidative stress. However, their fixed shapes make it difficult for them to fit irregular wound surfaces, and creating pores in these hydrogels can be complicated. Microgels offer a significant improvement in this area. Unlike traditional hydrogels, microgels can form porous scaffolds that allow air and moisture to reach the wound, which is essential for healing [189]. Microgels offer several advantages over hydrogels for wound treatment, particularly in chronic and diabetic wounds, due to their enhanced drug delivery precision, responsive nature, and versatility. Their small size and large surface area enable precise and controlled release of therapeutic agents directly at the wound site, improving treatment efficacy and reducing side effects. Unlike traditional hydrogels, which are effective for moisture retention but lack specificity, microgels can be engineered to respond to environmental stimuli, such as pH or temperature changes, ensuring that drugs are released precisely when needed. This dynamic release capability makes microgels particularly suitable for complex or challenging wounds that require targeted intervention. Additionally, the versatility of microgels allows them to be integrated into various application forms, offering customized solutions for different wound types. While hydrogels provide a moist healing environment, the responsive and customizable nature of microgels offers an advanced approach to wound care management. Yongyuan Kang et al. have introduced an innovative approach to enhance skin wound healing and reduce hypertrophic scar formation through the use of rod-shaped microgel scaffolds. These scaffolds are meticulously designed with larger surface areas and interconnected porous structures, optimizing gas transport and moisture retention to significantly improve wound oxygenation. This enhanced environment accelerates healing while minimizing the potential for scarring [190]. To address the challenges of creating effective, breathable wound dressings in moist environments, the researchers designed these microgel scaffolds to facilitate rapid gas exchange and fluid absorption. Crucially, they incorporate mesoporous zinc-doped hydroxyapatite (mZH) to support the structural integrity of the scaffolds and immobilize catalase (CAT), an enzyme with potent antioxidant properties. By integrating CAT directly into the microgel matrix, the system ensures immediate enzymatic action, allowing the enzyme to more efficiently interact with wound exudate and perform its antioxidative role. This direct integration offers a notable advantage over traditional systems that rely on slower enzyme release, thereby providing more immediate therapeutic effects, especially in acute wound conditions. These advancements highlight the potential of rod-shaped microgel scaffolds not only to accelerate healing processes but also to effectively alleviate the formation of hypertrophic scars, offering a promising avenue in skin tissue regeneration therapies, as shown in Figure 13.

**Figure 14:**
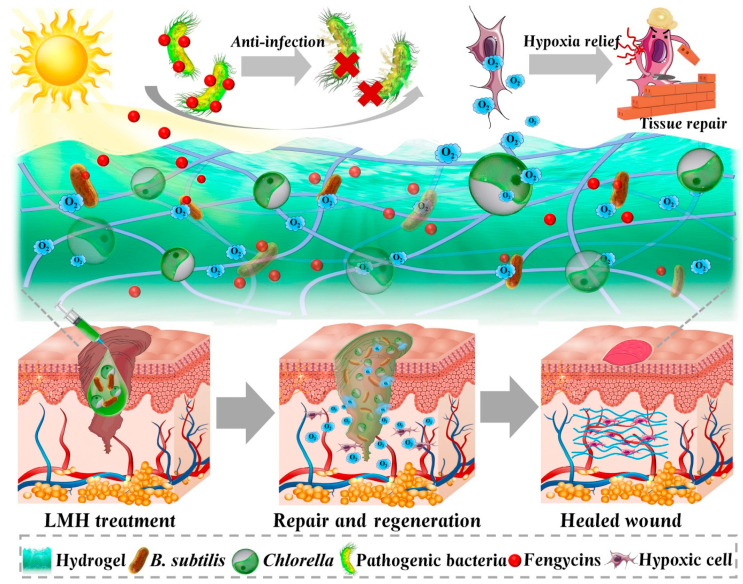
Schematic overview of LMH utilization for treating chronic wounds. The LMH combines oxygen-producing *Chlorella* and antimicrobial *B. subtilis* to fill the wound bed. *Chlorella* aids in oxygen generation and supports *B. subtilis*, which controls infections. This synergy promotes healing in infected diabetic wounds by addressing hypoxia and infection [191].

A similar investigation has been reported regarding living microecological hydrogels aimed at wound healing by introducing a living microecological hydrogel (LMH) designed to promote chronic wound healing by encapsulating functionalized *Chlorella* and *Bacillus subtilis*. The LMH facilitates continuous oxygen delivery and possesses anti-infection properties, effectively addressing the issues of hypoxia and infections commonly associated with hard-to-heal wounds. Composed of thermosensitive Pluronic F-127 and wet-adhesive polydopamine, the hydrogel remains in a liquid state at low temperatures but quickly solidifies and adheres tightly to the wound bed upon application. By optimizing the encapsulation ratio of the microorganisms, *Chlorella* can continuously produce oxygen, fostering the proliferation of *B. subtilis*, which, in turn, helps to eliminate pathogenic bacteria. Consequently, the LMH significantly enhances the healing process of infected diabetic wounds, highlighting its potential for practical clinical applications in wound management, as shown in Figure 14 [191].

#### Injectable Hydrogels for Wound Treatment

One study reveals that injectable, microporous gel scaffolds, constructed from annealed microgel building blocks, effectively overcome the challenge of premature hydrogel degradation, providing sustained support for tissue regrowth. By utilizing microfluidic fabrication, the scaffolds’ chemical and physical properties can be finely tuned, allowing for optimal conditions that promote cellular proliferation and network formation. In vitro experiments demonstrate that these scaffolds enable rapid cellular organization, with cells forming extensive three-dimensional structures within 48 h. Moreover, in vivo results show that the scaffolds promote swift cell migration, leading to rapid cutaneous tissue regeneration and new tissue structure formation within five days. The successful combination of microporosity and injectability in these scaffolds offers promising new avenues for tissue regeneration and formation, potentially revolutionizing therapeutic approaches in regenerative medicine [192]. One study presents a pioneering method for creating injectable microfiber–gel granules with bioceramic powders, resulting in microporous granular microgel–fiber hydrogels (MFgel). These are made by integrating fibers of hyaluronic acid and sodium alginate loaded with siRNA and bioglass particles. Compared to traditional hydrogels, MFgel’s micrometer-scale pores enhance cell adhesion and penetration. It also exhibits higher compressive strength and stability. In vivo studies show that MFgel supports cell and tissue infiltration in mice. In diabetic rats, it effectively manages inflammation, decreases MMP-9 expression, boosts angiogenesis, and accelerates wound healing, showing great potential for diabetic wound treatment [193]. Biomaterials based on natural product microgels are gaining attention for wound treatment due to their biocompatibility, biodegradability, and healing promotion. Formulated from biopolymers like alginate, chitosan, and gelatin, these microgels can be tailored for controlled drug release, moisture retention, and oxygen permeability. They may also incorporate bioactive compounds, such as antimicrobial agents and growth factors, to enhance healing and prevent infections. Overall, natural product microgels represent a promising and versatile approach to effective wound management. Campelo et.al.studied This study incorporated hydroalcoholic extract from Agaricus blazei Murill (EAb) into poly(vinyl alcohol) (PVA) and sodium alginate (SA) microgels, indicating physical cross-linking and exhibiting approximately 40% porosity and significant DPPH radical scavenging. Morphological analysis revealed pores containing EAb granules (0.3–0.6 µm). In vivo tests showed that, by day 14, mice treated with EAb-loaded microgels achieved over 99% wound contraction, increased epidermal and dermal thickness, higher type I collagen density, and reduced oxidative stress [194].

### 5.4. Microgels-Based Scaffolds for Tissue

**Figure 15:**
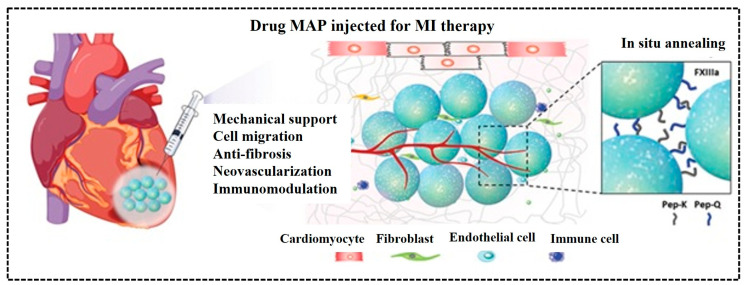
Microfluidic production of drug-releasing MAP (drugMAP) scaffolds for myocardial infarction (MI) therapy and their injection. Cardiac drugMAP scaffolds provide multiple benefits for heart repair through targeted drug delivery; reproduced with permission from Ref. [201]. Copyright © 2020, Wiley and Sons.

Microgels are innovative materials used in tissue engineering due to their ability to closely mimic the extracellular matrix and their highly tunable properties. These hydrogel-based particles can be engineered to have specific mechanical strengths, degradation rates, and bio-functional modifications, which are crucial for supporting cell adhesion, proliferation, and differentiation. In tissue engineering applications, microgels are used to create three-dimensional scaffolds that provide structural support for tissue regeneration. They facilitate the encapsulation and controlled release of bioactive agents such as growth factors, which can enhance cellular responses and tissue development. Microgels can be designed to be thermosensitive, pH-sensitive, or responsive to other physiological conditions, allowing for the localized and sustained release of therapeutics. In tissue engineering applications, microgels are used to create three-dimensional scaffolds that provide structural support for tissue regeneration. They facilitate the encapsulation and controlled release of bioactive agents, such as growth factors, which can enhance cellular responses and tissue development. Microgels can be designed to be thermosensitive, pH-sensitive, or responsive to other physiological conditions, allowing for the localized and sustained release of therapeutics. In recent advancements in tissue engineering, microgel assemblies have demonstrated exceptional potential in creating multistage structural microporous scaffolds that significantly enhance tissue regeneration and microtissue assembly. These assemblies provide nanoscale porosity from the material itself and microscale porosity from their structural assembly, effectively facilitating cell attachment, proliferation, and migration [195,196]. A noteworthy development by Wei et al. introduced polyhydroxyalkanoate open porous microgels (PHAOPMSs), which combine the benefits of microgels and scaffolds, providing ample open 3D space critical for cellular activities [197]. These microgel scaffolds have successfully regulated local inflammation, inhibited intervertebral disc degeneration, and promoted regeneration [198]. Beyond cell therapy, microporous scaffolds serve as effective platforms for establishing tumor models. He et al. utilized honeycomb-like porous GelMA microgel scaffolds to culture osteosarcoma cells (K7M2), demonstrating that the 3D cultured cells exhibited enhanced tumorigenicity, characterized by quicker tumor formation and more aggressive tumor growth, providing a potent microenvironment for tumor pathogenesis investigation and drug screening [199]. Additionally, the unique scaffold structure can promote vascular regeneration and bone formation, even in acellular conditions [200]. Expanding their utility, microgel-based systems are also being explored in regenerative medicine applications, such as drug delivery platforms aimed at reducing fibrosis and treating myocardial infarction. Jun Fang et al. investigated an innovative Injectable Drug-Releasing Microporous Annealed Particle (drugMAP) scaffold for myocardial infarction treatment. This system encapsulates hydrophobic drug-loaded nanoparticles into microgel building blocks through microfluidic manufacturing. By adjusting factors like nanoparticle hydrophilicity and pre-gel solution viscosity, they achieved a consistent and uniform encapsulation process [201]. This novel injectable, multimodal drug MAP hydrogel offers a promising therapeutic strategy for myocardial infarction, as illustrated schematically in their study (as shown in Figure 15).

These advancements underscore the versatile and transformative nature of microgel technologies within both regenerative medicine and tissue engineering fields, paving the way for novel therapeutic applications and complex tissue modeling. Currently, microgel assembly is being explored in vitro and in vivo for various tissue engineering applications, including neuronal, cardiac, vascular, cartilage, and skin tissues. In the quest to address long-term disabilities resulting from strokes, researchers have made groundbreaking discoveries. Nih et al. [202] explored the potential of peptide-modified HA microgels to transform the recovery landscape following a stroke. By injecting these innovative microgels into the stroke lesions of C57BL/6 male mice, they observed not just a decrease in the post-stroke inflammatory response but also the facilitation of the movement of astrocytes into the affected areas. This prevented the formation of obstructive glial scars and reduced the presence of reactive microglia, essential to fostering a healing environment. In turn, this led to enhanced vascularization around the infarct zone and encouraged the migration of neural progenitor cells, outperforming traditional nonporous hydrogel and stroke-only treatments. Meanwhile, in the realm of spinal injury, the work of Dumont et al. [203] took center stage. By crafting PEG-MAL microgels into tubular structures and bridges through photopolymerization, they developed an intervention suitable for C57BL/6J female mice with spinal cord injuries. The implanted constructs successfully bonded with the host tissue, thanks to infiltration by immune and supportive cells, opening a new chapter in spinal repair methods. Furthermore, addressing myocardial infarction, Mealy et al. [204] engineered a dual-component HA microgel assembly with protease-sensitive and stable microgels held together by unique guest–host interactions. This assembly boasted shear-thinning and self-healing capabilities, making it apt for therapeutic injections. Such advancements hint at a future where myocardial damage can potentially be minimized, maintaining ventricular integrity and functionality and forestalling heart failure. Each study represents a step forward in biomaterial science, promising innovative treatments for conditions once deemed irreparable. The comparative applications of hydrogels and synthesis methods are shown in Table 1.

## 6. Conclusions and Future Directions

The development and application of hydrogels and microgels represent significant advancements in biomedical engineering, particularly for drug delivery and wound healing. Their unique physicochemical properties including biocompatibility, hydrophilicity, and reversible phase transitions make them ideal for enhancing therapeutic agents’ performance. Hydrogels, with their three-dimensional network structure, enable a controlled, sustained release of drugs, optimizing therapeutic effects and minimizing systemic side effects, which is crucial in cancer therapy and chronic disease management. Conversely, microgels offer innovative targeted drug delivery solutions due to their tunable size, surface chemistry, and responsiveness to stimuli. This versatility is valuable in wound healing, where they can deliver growth factors or antimicrobials directly to the wound site, promoting healing and reducing infection risks.

The future approach to this study will be directed as follows:

1. Explore the development of multifunctional hydrogels and microgels with integrated diagnostic and therapeutic features, which could include imaging agents or sensors for real-time monitoring of treatment progression.
2. Utilize emerging technologies, such as 3D bioprinting, to create complex tissue scaffolds that mimic native tissue architecture, thereby enhancing strategies in regenerative medicine.
3. Optimize fabrication techniques to improve the uniformity and reproducibility of hydrogel and microgel production, employing advanced methods such as microfluidics and electrospinning for precisely engineered systems.
4. Investigate the interactions between hydrogels/microgels and biological systems, focusing on understanding immune responses and degradation pathways to ensure their safety and efficacy in clinical applications.
5. Continue interdisciplinary research to develop next-generation therapeutics, ultimately improving patient outcomes and advancing the field of regenerative medicine.

## Figures and Tables

**Table 1:** The summary of main findings on the properties and applications of hydrogel and microgel.

Parameter	Hydrogel	Microgel	References
Particle Size	Macro-scale	Submicron to micron-scale	[12,14]
Water Absorption	High water content to form gel-like consistency	Absorb water but as individual particles	[8,29,38,56]
Stimuli Response	Responds to changes in pH, temperature	Highly responsive to environmental stimuli	[48,49,139,152,153,190]
Applications	Drug delivery, tissue engineering	Cancer treatment, Antibacterial applicaction	[11,18,121,122,175,180]
Synthesis	Often involves cross-linking polymers in bulk	Colloidal synthesis methods, such as emulsion or precipitation polymerization	[42,44,46,152,153,174]
Responsiveness	Typically responds uniformly throughout the structure	Exhibits rapid and reversible responses due to small size and surface area	[14,44,175]
Recent Advancements	Incorporation of bioactive molecules, improved mechanical properties	Use of metallo-supramolecular cross-linkers for core-shell structures	[48,49,156,157]

## Data Availability

No new data were created or analyzed in this study.
